# Dysregulation of B Cell Activity During Proliferative Kidney Disease in Rainbow Trout

**DOI:** 10.3389/fimmu.2018.01203

**Published:** 2018-05-31

**Authors:** Beatriz Abos, Itziar Estensoro, Pedro Perdiguero, Marc Faber, Yehfang Hu, Patricia Díaz Rosales, Aitor G. Granja, Christopher J. Secombes, Jason W. Holland, Carolina Tafalla

**Affiliations:** ^1^Centro de Investigación en Sanidad Animal (CISA-INIA), Madrid, Spain; ^2^Fish Pathology Group, Institute of Aquaculture Torre de la Sal (IATS-CSIC) Castellón, Madrid, Spain; ^3^Scottish Fish Immunology Research Centre, Institute of Biological and Environmental Sciences, University of Aberdeen, Aberdeen, United Kingdom

**Keywords:** *Tetracapsuloides bryosalmonae*, proliferative kidney disease, rainbow trout, B cells, immunoglobulin T, immunoglobulin D, immunoglobulin M

## Abstract

Proliferative kidney disease (PKD) is a widespread disease caused by the endoparasite *Tetracapsuloides bryosalmonae* (Myxozoa: Malacosporea). Clinical disease, provoked by the proliferation of extrasporogonic parasite stages, is characterized by a chronic kidney pathology with underlying transcriptional changes indicative of altered B cell responses and dysregulated T-helper cell-like activities. Despite the relevance of PKD to European and North American salmonid aquaculture, no studies, to date, have focused on further characterizing the B cell response during the course of this disease. Thus, in this work, we have studied the behavior of diverse B cell populations in rainbow trout (*Oncorhynchus mykiss*) naturally infected with *T. bryosalmonae* at different stages of preclinical and clinical disease. Our results show a clear upregulation of all trout immunoglobulins (Igs) (IgM, IgD, and IgT) demonstrated by immunohistochemistry and Western blot analysis, suggesting the alteration of diverse B cell populations that coexist in the infected kidney. Substantial changes in IgM, IgD, and IgT repertoires were also identified throughout the course of the disease further pointing to the involvement of the three Igs in PKD through what appear to be independently regulated mechanisms. Thus, our results provide strong evidence of the involvement of IgD in the humoral response to a specific pathogen for the first time in teleosts. Nevertheless, it was IgT, a fish-specific Ig isotype thought to be specialized in mucosal immunity, which seemed to play a prevailing role in the kidney response to *T. bryosalmonae*. We found that IgT was the main Ig coating extrasporogonic parasite stages, IgT^+^ B cells were the main B cell subset that proliferated in the kidney with increasing kidney pathology, and IgT was the Ig for which more significant changes in repertoire were detected. Hence, although our results demonstrate a profound dysregulation of different B cell subsets during PKD, they point to a major involvement of IgT in the immune response to the parasite. These results provide further insights into the pathology of PKD that may facilitate the future development of control strategies.

## Introduction

Proliferative kidney disease (PKD) is a disease of major economic importance to salmonid aquaculture caused by the myxozoan parasite *Tetracapsuloides bryosalmonae* (1). Parasite malacospores are released from infected freshwater bryozoans, the invertebrate host of the parasite. Once in the water, the malacospores gain entry into the fish vascular system *via* the gills (2) and migrate to different organs, the kidney being the main focus of parasite development and proliferation (1). The teleost kidney is the equivalent of mammalian bone marrow as it is the largest site of hematopoiesis and the organ responsible for B cell development (3). In addition, it has also been reported to function as a secondary immune organ (4). When the water temperature rises above 15°C, the kidney responds to the presence of *T. bryosalmonae* extrasporogonic stages with a strong hyperplastic response leading to the regression of urinary tissues and anemia due to reduced erythropoietin production by cells within each nephron (5). Consequently, the fish are much more susceptible to secondary infections, and mortalities up to 95–100% can be reached (1). Below 15°C, the host develops a milder immune response to the parasite that is associated with fewer clinical signs and almost no mortality (6, 7).

Proliferative kidney disease has been defined as an immunopathological condition mediated by an exacerbated host leukocyte response to the parasite (7–9). This induced response seems to be mediated by lymphocytes, which increase in percentage during the course of the disease while granulocyte populations sharply decrease (7, 8). Several transcriptional studies performed during the course of PKD have revealed the regulation of an important number of genes related to Th functions such as IL-4/13A, GATA3, or IL-10 (7, 9). However, further studies on how T cells are affected by the parasite have not been performed and this should also be addressed in the future. In addition, these transcriptional studies have provided evidence that points toward a profound dysregulation of B cells in the kidney during PKD. For example, Gorgoglione et al. (9) analyzed the expression profile of a wide panel of immune molecules in rainbow trout (*Oncorhynchus mykiss*) following a natural exposure to the parasite and found that immunoglobulin (Ig) transcription was strongly upregulated in significant correlation to the stage of kidney pathology (9). Similarly, Bailey et al. (7) established that the transcription of secreted IgM (sIgM) and B cell-related genes such as Blimp1 were strongly induced after an experimental infection with the parasite at 15°C but not at 12°C (7). Finally, a recent study performed by our group demonstrated that the cytokines of the BAFF/APRIL family, known to play a major role in B cell differentiation and survival in mammals, were significantly modulated, along with their receptors, by the parasite and correlated with the transcriptional levels of different Ig isotypes (10).

Fish only express three Ig classes, namely, IgM, IgD, and IgT (designated as IgZ in some fish species) (11). As in mammals, most B cells found in central lymphoid tissues such as spleen and peripheral blood express both IgM and IgD on the cell surface (12). Similar to the situation in mammals, these cells seem to lose IgD during their differentiation to plasmablasts/plasma cells (12, 13). In addition, as reported in humans in specific mucosal surfaces such as the upper respiratory tract (14), B cells exclusively expressing IgD on the cell surface have also been reported in rainbow trout gills (15) and catfish blood (16), although their precise role is still unknown. Finally, IgT, a teleost-specific Ig, is expressed on the surface of a distinct linage of B cells (17, 18). IgT^+^ B cells constitute around 51% of all B cells in the intestinal mucosa, whereas they only represent around 18–27% of all B cells in central lymphoid organs such as spleen, kidney, or peripheral blood (18). This, together with the fact that IgT was the major responder to *Ceratonova shasta* (a myxozoan parasite with intestinal tropism) in mucosal compartments while IgM was the main Ig responding systemically to the parasite led the authors to hypothesize that IgT is specialized in mucosal immunity in teleost fish (18). Further studies supported this hypothesis in describing a similar role of IgT in gills (19) and skin (20) in response to *Ichthyophthirius multifiliis*, a protozoan parasite with strong tropism for both mucosal tissues. Nevertheless, evidence for IgT responses outside the mucosal compartments has also been reported suggesting that IgT might also play an important role in fish systemic responses. For example, Zhang et al. already described a similar capacity of IgM^+^ and IgT^+^ B cells in the kidney to proliferate and respond to *Vibrio anguillarum* (18). Furthermore, IgT responses were found, in addition to IgM responses, in the spleen of rainbow trout exposed to a systemic viral infection (21) and in the muscle of DNA vaccinated fish (22).

Although fish are able to mount specific antibody responses against a wide range of pathogens, it is generally accepted that the lack of specialized structures where B cells can closely interact with T-helper cells such as germinal centers (GCs) and lymph nodes strongly conditions the immune response generated in this animal group (23). In mammals, three different mechanisms have been described to generate antibody diversity, as the basis of a specific humoral immune response. Before exposure to an antigen, the initial generation of a broad antibody repertoire is achieved early in B cell development by rearrangement of the V, D, and J gene segments to produce Igs with unique Ig heavy- and light-chain variable regions (IGHV and IGLV) (23). A second strategy to increase the Ig repertoire is through junctional diversity, a number of different processes through which different sizes are generated in the heavy-chain sequences by imprecise V(D)J recombination. Terminal deoxynucleotidyl transferase (TdT) is one of the enzymes responsible for the generation of this junctional diversity, through the addition of non-templated (N) nucleotides to the single-strand DNA ends (24). Finally, during B cell differentiation, the genes encoding the variable domains of the heavy and light chains undergo a high degree of point mutations through a process designated as somatic hypermutation (SHM). SHM results in the increased diversity of the antibody pool after which only the cells with higher affinity are selected by follicular antigen presenting cells. This diversification is critical for the generation of an adequate specific immune protection (25) and is mediated by the enzyme activation-induced deaminase (AID), which also plays a role in class switch recombination (CSR), the mechanism through which B cells replace the constant region of the heavy-chain associated with the variable region to produce Ig isotypes with a higher affinity than IgM, such as IgG, IgA, or IgE (26). To date, no CSR has been reported in fish and although all the elements to induce the variability of the B cell repertoire have been identified, fish fail to induce substantial increases in the affinity of their specific antibodies (27).

In this study, following the transcriptional evidence that demonstrated an increase of IgM and IgT in rainbow trout kidney with increasing clinical disease (9), we have undertaken an in depth analysis of B cell and Ig responses in naturally PKD-infected rainbow trout exhibiting early to advanced clinical disease. We have demonstrated that all three Ig isotypes are increased at the protein level in the kidney in response to the parasite and that four different B cell subsets coexist in the infected kidney according to the expression of different Ig isotypes (namely, IgM^+^IgD^+^, IgM^+^IgD^−^, IgD^+^IgM^−^, and IgT^+^ cells). In addition, the repertoire analysis of the three Ig subtypes in fish with no clinical signs of disease in comparison to fish with an evident pathology revealed significant changes in VH family usage, clonal expansion, and mutation rate that suggested the involvement of all three Igs in the response to the parasite. These results constitute the first evidence of IgD regulation in response to a pathogen in teleost fish, providing new data to further understand the role of this Ig. Nevertheless, multiple factors point to a prevailing role for IgT during clinical PKD. IgT was the predominant Ig coating the parasite; IgT^+^ B cells were found to be actively proliferating in advanced stages of the disease, whereas the percentage of IgM^+^ and IgD^+^ proliferating cells was much lower; and the IgT repertoire was altered to a greater extent than that of IgM and IgD. Hence, our results show a predominant role of IgT in the immune and pathogenic response to PKD, which provides further evidence of IgT function outside mucosal compartments in fish. Furthermore, the data presented constitutes valuable information for the generation of novel treatments against PKD.

## Materials and Methods

### Fish Sampling

Two groups of rainbow trout from the same egg source (50–100 g each) were used in this study, as described previously (9). For this, one group of rainbow trout was moved in early April to a commercial trout farm with a history of PKD outbreaks located in Southern England. Clinical signs of the disease were first seen in these fish in early June. One week before the sampling (late July), the second (parasite-naïve) group was moved to this fish farm, so that sampling of both groups was undertaken at a water temperature of 15–16°C. At this point, the fish that had been exposed to the parasite from April exhibited kidney pathology ranging from early to advanced clinical stages (kidney swelling grades 1–4), as established using the kidney swelling index system described by Clifton-Hadley et al. (28), whereas fish that were moved to the farm 1 week before the sampling showed no clinical disease (grade 0 kidney swelling). The presence of *T*. *bryosalmonae* in parasite-infected fish was confirmed by histological examination of posterior kidney smears and by PCR, as described previously (9). In all fish sampled, approximately 100 mg of kidney tissue was removed immediately below the dorsal fin, the kidney area associated with the onset of the clinical disease. Tissue samples were placed into 1 ml of RNA-later (Sigma, St. Louis, MO, USA), kept at 4°C for 24 h and stored at −80°C before RNA and protein extraction. Tissue sections (ca. 4 mm × 3 mm × 3 mm) were excised from the trunk kidney and fixed in 5 ml ice-cold 4% paraformaldehyde (Sigma) for 24 h at 4°C, rinsed repeatedly with ice-cold phosphate-buffered saline (PBS) (10 min at 4°C per rinse), and maintained at 4°C in 70% ethanol until processed for histological analysis.

### Ig Immunohistochemical Detection

Kidney portions of parasitized fish fixed in 4% paraformaldehyde were processed for paraffin embedding following routine histological procedures. Thereafter, 4 μm-thick tissue sections were mounted on Superfrost Plus slides (Menzel-Gläser). Endogenous peroxidase was quenched with 0.3% hydrogen peroxide, and antigens were retrieved by heating in Tris–EDTA buffer (10 mM Tris base, 1 mM EDTA, pH 9) in a microwave oven for 5 min at 800 W and 5 min at 450 W. Thereafter, non-specific binding was blocked with 5% bovine serum albumin (BSA) in Tris-buffered saline (TBS). Slides were then incubated with specific mouse mAbs recognizing the different Igs. In the case of the anti-trout IgM (20 µg/ml) (29) and the anti-trout IgD (15 µg/ml) (30), this incubation step was performed for 1 h at room temperature (RT), whereas in the case of the anti-mouse IgT (10 µg/ml) (29) the incubation was performed overnight at 4°C. Then, a secondary anti-mouse IgG antibody conjugated with horseradish peroxidase was added and visualized with 3,3′-diaminobenzidine tetrahydrochloride chromogen (EnVision+ System/HRP, Dako). Eventually, sections were counterstained with Gill’s hematoxylin, dehydrated and mounted in DPX (di-*N*-butyl-phthalate in xylene). Images were acquired with a Leica DFC320 digital camera connected to a Leica DM LS optic microscope. To establish the coating of parasites with the different Ig isotypes, the presence and absence of IgM-, IgD-, and IgT-immunoreactivity in 100 parasites was recorded from different individual fish (*n* = 6), and statistically significant differences between IgM, IgD, and IgT parasite coating were calculated using a one-way ANOVA followed by a two-tailed Student’s *t*-test.

### RNA and Protein Extraction

Kidney samples were homogenized in 1.5 ml Tri-reagent (Sigma), and tissue debris removed by centrifugation at 12,500 × *g* for 10 min at 4°C. Initially, RNA and DNA were separated from protein by chloroform phase separation. Total RNA was extracted following the manufacturer’s instructions, dissolved in TE buffer (pH 8.0) and stored at −80°C until use. Purified RNA was quantified using a Nanodrop spectrophotometer (NanoDrop Technologies, Wilmington, DE, USA) and reverse transcribed into cDNA as described previously (9).

In parallel, 2.25 ml of ice-cold isopropanol was added to the remaining phenol–ethanol supernatant, incubated at RT for 10 min and centrifuged at 12,000 × *g* for 10 min at 4°C. Protein pellets were washed twice in 3 ml of 0.3 M guanidine hydrochloride in 95% ethanol, incubated for 20 min at RT and centrifuged at 7,500 × *g* for 5 min at 4°C. Protein was resuspended in 2 ml absolute ethanol, incubated for 20 min at RT, and pellets solubilized in the presence of urea and sodium dodecyl sulfate (SDS) (400 µl per pellet containing 8 M urea, 1% SDS in Tris–HCl, pH 8.0). To facilitate solubilization, protein pellets were sonicated repeatedly on ice and debris removed by centrifugation (10,000 × *g* for 10 min at 4°C).

### Western Blot Analysis

Protein concentration in kidney lysates was calculated using the bicinchoninic acid assay (Thermo Scientific) and BSA as a protein standard. Ten micrograms of each lysate were denatured with a 4× loading buffer containing SDS. The mixture was boiled at 100°C for 5 min and loaded onto a denaturing 10% SDS-PAGE gel (Mini-Protean TGX gels, Bio-Rad). Proteins were then transferred onto a polyvinylidene difluoride membrane (Trans-Blot Turbo Transfer Pack, Bio-Rad) using a semi-dry transfer system (Bio-Rad). Membranes were blocked in PBS containing 5% skimmed milk for the detection of IgM, IgD, and IgT and in PBS containing 2% BSA for the detection of α-tubulin. Following this blocking step, membranes were incubated with the corresponding antibody: anti-IgM and anti-IgD were used at 5 µg/ml, anti-IgT at 0.35 µg/ml, and anti-α-tubulin at 0.1 µg/ml (Abcam). Primary antibodies were incubated in blocking solution overnight at 4°C. Membranes were then washed with PBS containing 1% Tween-20 and incubated with a goat anti-mouse IgG-HRP conjugate (GE Healthcare Life Sciences) for 1 h at RT. Finally, the resulting bands were visualized using the ECL system (GE Healthcare Life Sciences). Band intensities were quantified by optical densitometry and normalized against α-tubulin using ImageJ software (National Institutes of Health).

### Immunofluorescence and Confocal Microscopy

A double immunofluorescent detection of IgD and IgM was performed using 4 µm sections of paraffin-embedded trunk kidney samples. For this, antigen retrieval, blocking of the non-specific binding and incubation with the primary anti-IgD antibody were performed as described earlier for the immunohistochemical detection of IgD. Thereafter, sections were incubated with a secondary AlexaFluor^®^488 anti-mouse antibody (Life Technologies). A second blocking step was conducted by incubating the sections with TBS containing 5% BSA overnight at 4°C. Thereafter, a biotinylated anti-IgM mAb was added followed by incubation with Streptavidin AlexaFluor^®^647 (Life Technologies). Sections were then counterstaining with DAPI (1 µg/ml, Sigma), incubated with 0.3% Sudan black B in 70% ethanol for 10 min to remove tissue autofluorescence, rinsed with TBS and mounted with Fluoromount (Sigma). Immunoreactive cells were visualized with a confocal laser-scanning microscope (Zeiss LSM 880) with Zeiss Zen software.

To determine the percentage of proliferating IgM^+^, IgD^+^, or IgT^+^ B cells at different stages of clinical disease, 4 µm sections of paraffin-embedded trunk kidney samples were also used. In these assays, the different mouse monoclonal antibodies directed against IgM, IgD, and IgT were co-incubated with an antibody directed against the proliferating cell nuclear antigen (PCNA), an intracellular molecule whose expression and synthesis is linked with cellular proliferation (31). Antigen retrieval and blocking of the non-specific binding were performed as described earlier for the immunohistochemical detection of the Ig isoforms. Tissues were then incubated with the corresponding primary antibody (anti-IgM 20 µg/ml; anti-IgD 15 µg/ml; and anti-IgT 10 µg/ml). Incubation with a secondary goat anti-mouse IgG1 antibody conjugated with AlexaFluor^®^488 (ThermoFisher) was followed by a further incubation with a mouse IgG2 anti-PCNA antibody conjugated with AlexaFluor^®^647 (BioLegend) and counterstained with DAPI (1 µg/ml, Sigma). Tissue autofluorescence was then blocked by incubation with 0.3% Sudan black B in 70% ethanol for 10 min, sections were rinsed with TBS and mounted with Fluoromount. Immunoreactive cells were visualized, and images acquired with a confocal laser-scanning microscope. Tissue images were then analyzed in 10 digital fields at 400× magnification of each tissue section with Zeiss Zen and ImageJ software packages. Statistically significant differences of proliferating and non-proliferating IgM-, IgD-, and IgT-immunoreactive cells between grade 0 and grade 2 parasite-exposed fish were analyzed by one-way ANOVA followed by a two-tailed Student’s *t*-test. Data that failed the normality or equal variance test were analyzed with Kruskal–Wallis one-way ANOVA on ranks followed by Dunn’s method (*P* < 0.05).

### Analysis of AID and TdT Transcription

As an initial estimate of the level of expansion and differentiation of the B cell response that takes place during the course of PKD, we analyzed the levels of transcription of AID and TdT in kidney samples. For this, real-time PCR was performed in a LightCycler 96 System instrument (Roche) using FastStart Essential DNA Green Master reagents (Roche) and specific primers (shown in Table S1 in Supplementary Material). Amplification to determine the levels of transcription of the different Ig isoforms was performed in parallel in the same samples to determine whether correlations could be established. The efficiency of the amplification was determined for each primer pair using serial 10-fold dilutions of pooled cDNA, and only primer pairs with efficiencies between 1.95 and 2 were used. Each sample was measured in duplicate under the following conditions: 10 min at 95°C, followed by 40 amplification cycles (30 s at 95°C and 1 min at 60°C). The expression of individual genes was normalized to that of trout EF-1α, and expression levels calculated using the 2^−ΔCt^ method, where ΔCt is determined by subtracting the EF-1α value from the target Ct as described previously (32, 33). EF-1α was selected as reference gene according to the MIQE guidelines (34) given that no statistical differences were detected among Ct values obtained for EF-1α in the different samples. Negative controls with no template and *minus-*reverse transcriptase (−RT) controls were included in all experiments. A melting curve for each PCR was determined by reading fluorescence at every degree between 60 and 95°C to ensure only a single product had been amplified. Statistical analyses were performed by one-way ANOVA followed by a two-tailed Student’s *t*-test.

### Repertoire Analysis

To study how clinical disease altered the IgM, IgD, and IgT repertoire, cDNAs from four representative fish with grade 0 and four with grade 2 were used. These cDNAs were amplified using a forward primer specific for a subgroup of the IGHV genes together with a reverse primer specific for IGHM, IGHD, or IGHT genes (C_μ_, C_δ_, or C_τ_) previously designed by Castro et al. (21) (Table S2 in Supplementary Material). Mix reactions for the PCR were as follows: 1 µl of cDNA was used as template using 0.2 mM of each dNTP, 0.2 mM of each primer, and 0.03 U/μl DNA polymerase (Biotools) in 1× reaction buffer containing 2 mM MgCl_2_. The PCR was programmed as follows: an initial step of 95°C for 5 min followed by 40 cycles of 95°C for 45 s, 60°C for 60 s, and 74°C for 45 s and a final extension step of 74°C for 10 min. Negative controls without cDNA were also included.

An 8 µl aliquot of each PCR was mixed with 2 µl of loading buffer and loaded into a 1% agarose gel stained with SYBR Safe for visualization (Figure S1 in Supplementary Material). In parallel, 2 µl aliquots of each PCR product belonging to the same individual were pooled together. DNA concentrations were measured using the QuBit DNA quantification system (Invitrogen), and the quality was checked using an Agilent 2100 Bioanalyzer (Agilent Technologies). One library per individual was constructed with the TruSeq DNA PCR-Free Library Prep Kit (Illumina, San Diego, CA, USA) according to the manufacturer’s protocol. Libraries were pooled together, and paired-end sequencing was performed on an Illumina MiSeq with a MiSeq Reagent Kit v3 (2 × 300 cycles) cartridge (Illumina, San Diego, CA, USA).

### Sequence Analysis

Raw data were demultiplexed and sequencing adapters and barcodes were removed from the sequences by the MiSeq Analysis pipeline. A quality filter of phred base quality ≥20 was applied to reads. The first 20 nt from reverse primers used in PCRs were used as barcode for the identification of 3′ ends corresponding to the constant gene. Reads from R1 or R2 that perfectly matched a primer sequence were classified into the corresponding isotypes (IgM, IgD, or IgT) using the FASTQ/A Barcode splitter tool (http://hannonlab.cshl.edu/fastx_toolkit/). The opposite paired end reads, corresponding to the 5′ end of PCR products, were extracted with FASTQ interlacer tool implemented in Galaxy (35). The paired forward and reverse reads were then merged using PEAR software (36) with a minimum overlap size of 12 nt. When no overlap was detected, reads corresponding to the 5′ end were retained and included together with merged reads in successive analysis.

Sequences for each isotype from the eight individuals were compared with available information from *O. mykiss* contained in the International Immunogenetics information system databases (37) using IMGT/HighV-QUEST tool (38). Immune repertoire and SHM&CSR pipelines from Antigen receptor Galaxy tool (39) were used to analyze V(D)J usage, complementarity-determining region 3 (CDR3) characteristics and SHM. To estimate the PCR polymerase and sequencing error ratio, a short fragment without polymorphism covering the first 50 bp from the reverse primer of the IgM constant region was selected. The selected region was mapped to the reference germline sequence from IMGT using BWA (40). The error ratio was calculated as the number of mismatches regarding the reference sequence.

## Results

### Ig Production at Different Stages of Clinical Disease

**Figure 1:**
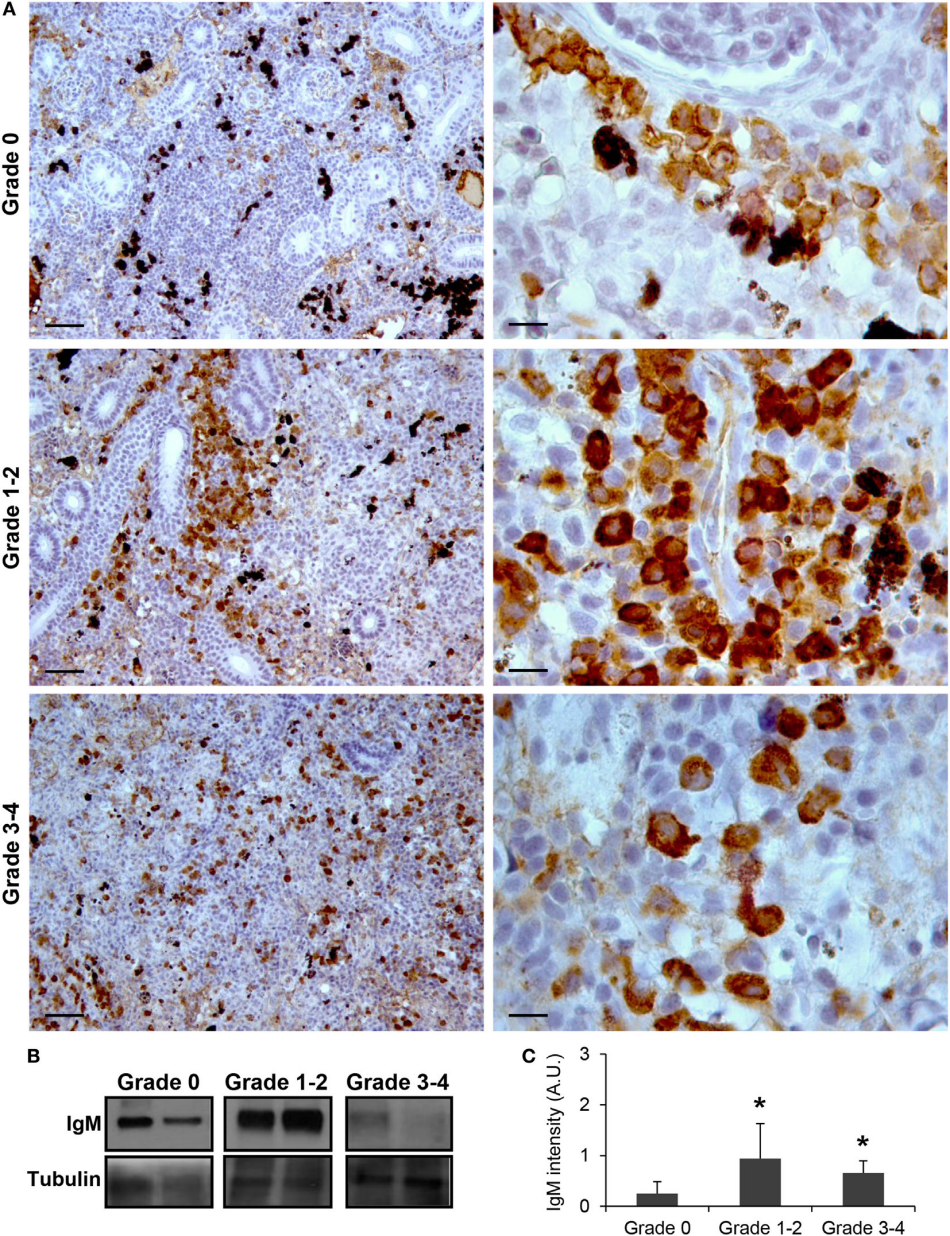
IgM production increases during proliferative kidney disease infection. **(A)** Immunohistochemical detection of IgM^+^ cells in the kidney of parasite-infected (swelling grades 1–4) and parasite-naïve (swelling grade 0) fish. Representative images for fish with grade 0, grade 1–2, and grade 3–4 are shown at 20× magnification (left images, scale bars = 50 µm) and at 100× magnification (right images, scale bars = 10 µm). IgM production in kidney samples from each fish group was analyzed by Western blot. **(B)** Results from two representative individuals in each group are shown. **(C)** Abundance of IgM was quantified by optical densitometry and normalized against α-tubulin. Mean values in arbitrary units (A.U.) + SEM are shown (*n* = 4). Asterisks (*) denote significant differences (*P* < 0.05) between values obtained in grade 1–2 or grade 3–4 and grade 0 kidneys (analyzed by a two-tailed Student’s *t*-test).

As observed in Figure 1A, IgM^+^ B cells were detected in the kidney of parasite-naive fish exhibiting no signs of PKD (grade 0) with a pattern similar to that previously reported for uninfected fish (41). In these animals, IgM^+^ B cells were scattered throughout the kidney stroma. Most of these cells showed a lymphocyte-like morphology, that is, round or slightly ovoid cells with a large, round and centrally located nucleus, thus resulting in a high nucleus:cytoplasm ratio. A strong increase in IgM positivity (defined as intensity of immunoreactivity) was observed in fish that exhibited grade 1–2 swelling grade (Figure 1A). In these fish, the total number of IgM^+^ B cells found in the kidney increased and these cells were mostly grouped in large cell clusters. Furthermore, the positivity of the individual cells was higher than that observed in grade 0 fish, suggesting higher levels of IgM per cell (Figure 1A). Interestingly, cells with a larger cytoplasm and an eccentric nucleus were more frequently observed in grade 1–2 fish, pointing to the potential differentiation of some IgM^+^ B cells to plasmablasts/plasma cells at this stage. A larger variability in the level of IgM reactivity was observed in kidney samples in fish with the highest level of clinical disease (grade 3–4). However, in most of these fish, the reactivity was reduced to levels either similar to those found in grade 0 fish or slightly higher (Figure 1A). Despite this, the reactivity of individual cells remained high when compared with that of IgM^+^ B cells from grade 0 fish (Figure 1A). All these results obtained by immunohistochemistry were further confirmed by Western blot analysis (Figures 1B,C). A significant increase in IgM reactivity was observed in both grade 1–2 and grade 3–4 kidney samples in comparison with that obtained in grade 0 kidney samples (Figure 1C).

**Figure 2:**
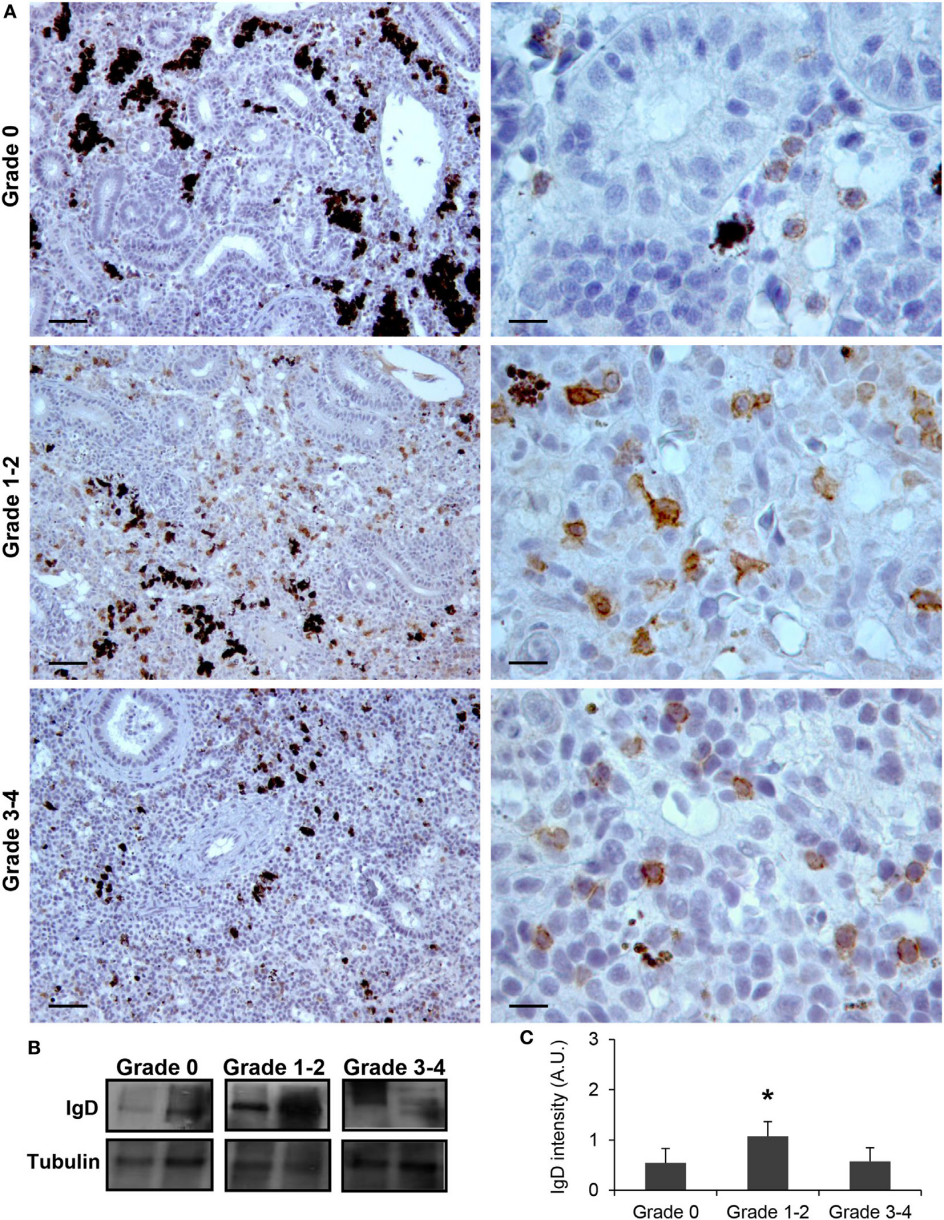
IgD production increases during proliferative kidney disease infection. **(A)** Immunohistochemical detection of IgD^+^ cells in the kidney of parasite-infected (swelling grades 1–4) and parasite-naïve (swelling grade 0) fish. Representative images for fish with swelling grades 0, 1–2, and 3–4 are shown at 20× magnification (left images, scale bars = 40 µm) and at 100× magnification (right images, scale bars = 10 µm). IgD production in trunk kidney samples from each fish group was analyzed by Western blot. **(B)** Results from two representative individuals in each group are shown. **(C)** Abundance of IgD was quantified by optical densitometry and normalized against α-tubulin. Mean values in arbitrary units (A.U.) + SEM are shown (*n* = 4). Asterisks (*) denote significant differences (*P* < 0.05) between values obtained in grade 1–2 or grade 3–4 and grade 0 kidneys (analyzed by a two-tailed Student’s *t*-test).

**Figure 3:**
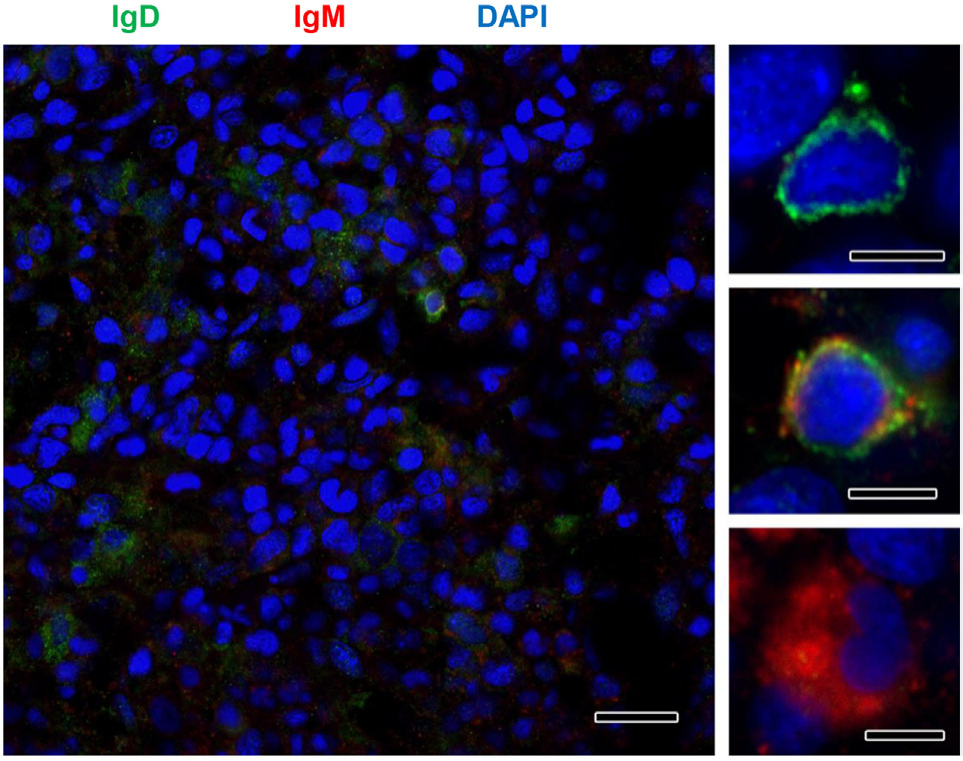
IgM and IgD detection in rainbow trout kidney infected with *Tetracapsuloides bryosalmonae*. IgD (green) and IgM (red) were immunolabeled in grade 2 kidney sections and counterstained with DAPI (blue). Details show the presence of immunoreactive IgD^+^/IgM^−^, IgD^+^/IgM^+^, and IgD^−^/IgM^+^ B cells. Scale bar in left hand image = 20 µm; scale bars in right hand images = 5 µm.

Although no significant increases in the levels of transcription of IgD had been reported in the kidney in response to PKD (9), our protein analysis demonstrated a significant increase in the levels of IgD protein with increasing clinical PKD, confirmed by both immunohistochemistry (Figure 2A) and Western blot (Figures 2B,C). While a small number of IgD^+^ B cells were found scattered through the kidney stroma in grade 0 fish, the number of IgD^+^ B cells increased along with their individual reactivity, especially in grade 1–2 fish (Figure 2A). Through Western blot analysis, we established that the level of IgD expression in grade 1–2 kidneys was significantly higher than that of grade 0 kidneys (Figure 2C); however, these differences were no longer significant in grade 3–4 kidney samples due to the large variability (Figure 2C). In addition, in contrast to what occurred with IgM^+^ B cells, the cells remained scattered, and no large clusters were observed at this stage (Figure 2A). The different distribution and number of IgD^+^ B cells when compared with IgM^+^ B cells observed in preparations from the same fish strongly suggests that not all cells that were visualized are IgM^+^IgD^+^ B cells, and that single positive cells for either of the two Igs should be present in the kidney. To confirm this point, a double immunofluorescence staining was performed using anti-IgM and anti-IgD in kidney sections from grade 2 fish. In these preparations, three different types of B cells were identified according to their pattern of IgM/IgD expression (Figure 3). These included IgM^+^IgD^+^ (that may correspond to non-differentiated B cells) and IgM^+^IgD^−^ cells (possibly IgM plasmablasts/plasma cells) as well as an IgD^+^IgM^−^ population (Figure 3) similar to the one previously reported in rainbow trout gills (15) and catfish blood (16).

**Figure 4:**
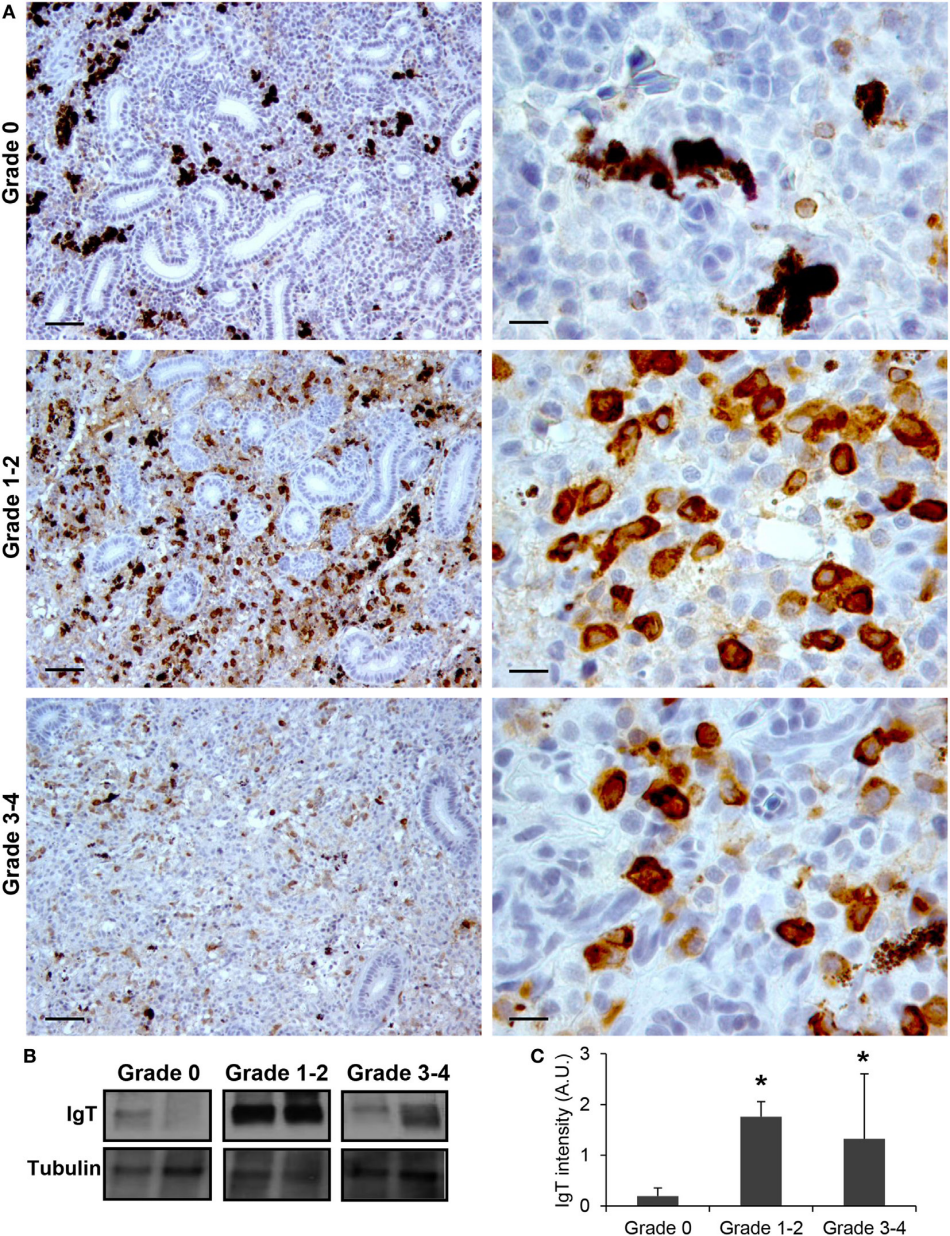
IgT production increases during proliferative kidney disease infection. **(A)** Immunohistochemical detection of IgT^+^ cells in the kidney of parasite-infected (swelling grades 1–4) and parasite-naïve (swelling grade 0) fish. Representative images for fish with swelling grades 0, 1–2, and 3–4 are shown at 20× magnification (left images, scale bars = 40 µm) and at 100× magnification (right images, scale bars = 10 µm). IgT production in kidney samples from each fish group was analyzed by Western blot. **(B)** Results from two representative individuals in each group are shown. **(C)** Abundance of IgT was quantified by optical densitometry and normalized against α-tubulin. Mean values in arbitrary units (A.U.) + SEM are shown (*n* = 4). Asterisks (*) denote significant differences (*P* < 0.05) between values obtained in grade 1–2 or grade 3–4 and grade 0 kidneys (analyzed by a two-tailed Student’s *t*-test).

Finally, we also studied the levels of IgT protein expression in the kidneys of fish from grade 0 to 4. Although IgT has been postulated as an Ig specialized in mucosal immunity, the presence of IgT^+^ B cells in the kidney has already been reported (18). In this study, a considerable number of IgT^+^ B cells with a lymphocyte-like morphology were found scattered throughout the kidney stroma in fish with no signs of clinical disease (Figure 4A). In fish exhibiting grade 1–2 swelling scores the positivity of IgT in the kidney was massively increased (Figure 4A). Although some of these cells appeared to be grouped together, no large cell clusters such as those observed for IgM were apparent (Figure 4A). The IgT reactivity decreased in kidney samples from grade 3–4 fish, although the number of IgT^+^ B cells was still higher than that observed in fish with no signs of disease. As for IgM, the positivity of individual cells was higher in animals with swelling grades from 1 to 4, suggesting that some IgT^+^ B cells differentiate to IgT-producing plasmablasts/plasma cells. Supporting this hypothesis, the identification of IgT^+^ cells with plasmablast morphology was evident in these fish (Figure 4A). All these immunohistochemistry results were confirmed by Western blot analysis (Figures 4B,C).

### IgT Is the Main Ig Isotype Coating *T. bryosalmonae*

**Figure 5:**
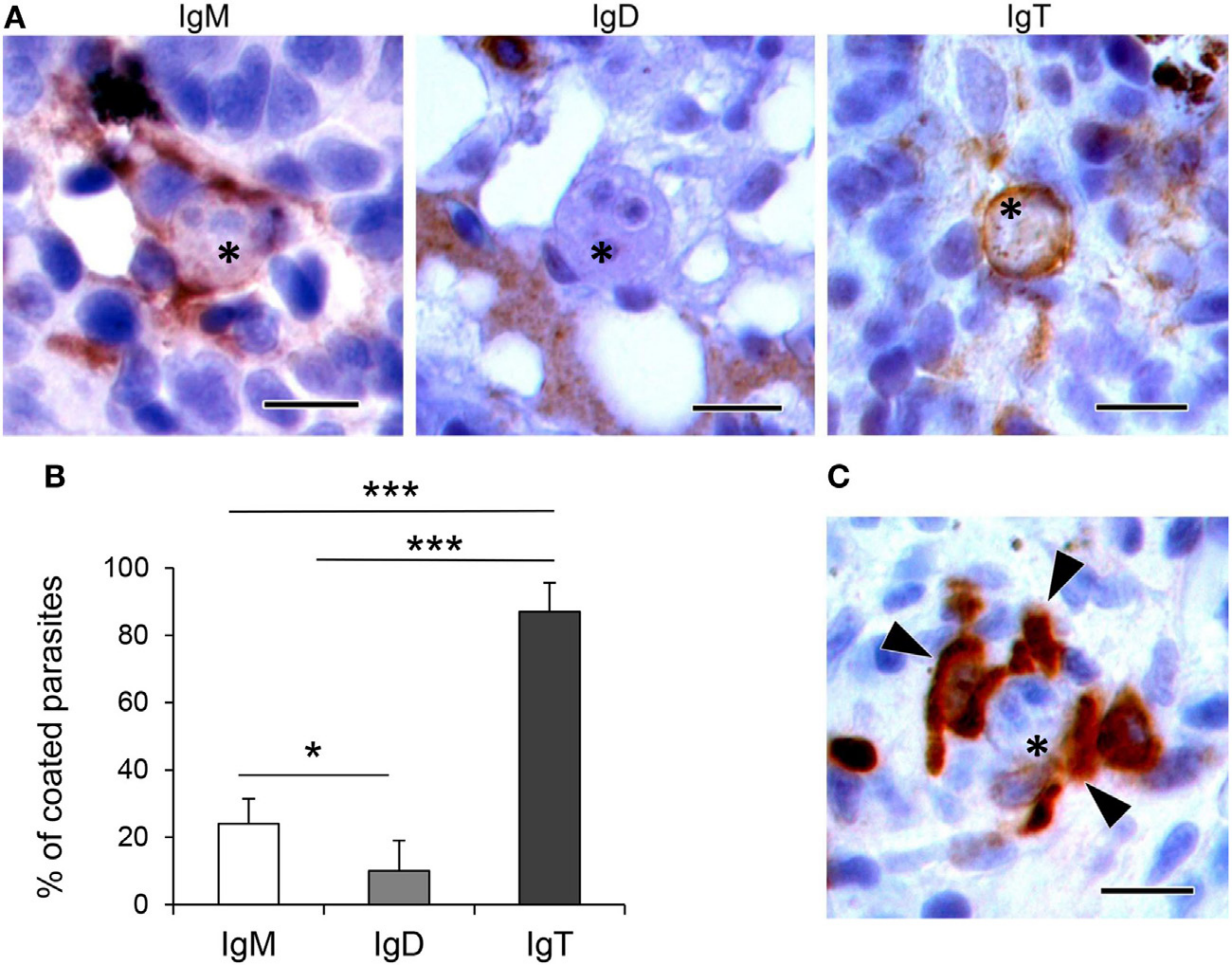
Coating of *Tetracapsuloides bryosalmonae* with host Igs. **(A)** Representative images showing parasites (indicated with asterisks) in the trunk kidney of rainbow trout coated with IgM, IgD, and IgT. Note the strong IgM and IgT immunoreactivity surrounding parasite stages, compared with low IgD immunoreactivity. Scale bars = 10 µm. **(B)** Mean percentage of parasites coated with each Ig. The presence and absence of IgM, IgD, and IgT immunoreactivity was recorded in 100 parasites from different individual fish with swelling grades from 1 to 4 (*n* = 6), and statistically significant differences between parasite coating with the different Ig calculated by a one-way ANOVA followed by two-tailed Student’s *t*-test (*P < 0.05 and ***P < 0.005). **(C)** Representative image showing several IgT^+^ cells (indicated with arrowheads) in close contact with a parasite (indicated with an asterisk). Scale bar = 10 µm.

To determine whether the different Ig isotypes were coating *T. bryosalmonae*, we evaluated the percentage of parasites with strong surface positivity for the different Ig isotypes in the respective immunohistochemistry preparations (from fish with swelling grades of 1–4). As observed in Figures 5A,B, we found that 24 and 10% of the parasites visualized in the preparations stained with anti-IgM or anti-IgD were coated by IgM and IgD, respectively. However, approximately 87% of the parasites visualized in the preparations stained with anti-IgT were coated with IgT. Furthermore, the presence of several IgT^+^ B cells surrounding the parasite, in close contact with it, was often evident in these preparations (Figure 5C). These results indicate that IgT is the main Ig isotype coating *T. bryosalmonae* in the kidney.

### B Cell Proliferation at Different Stages of Clinical Disease

**Figure 6:**
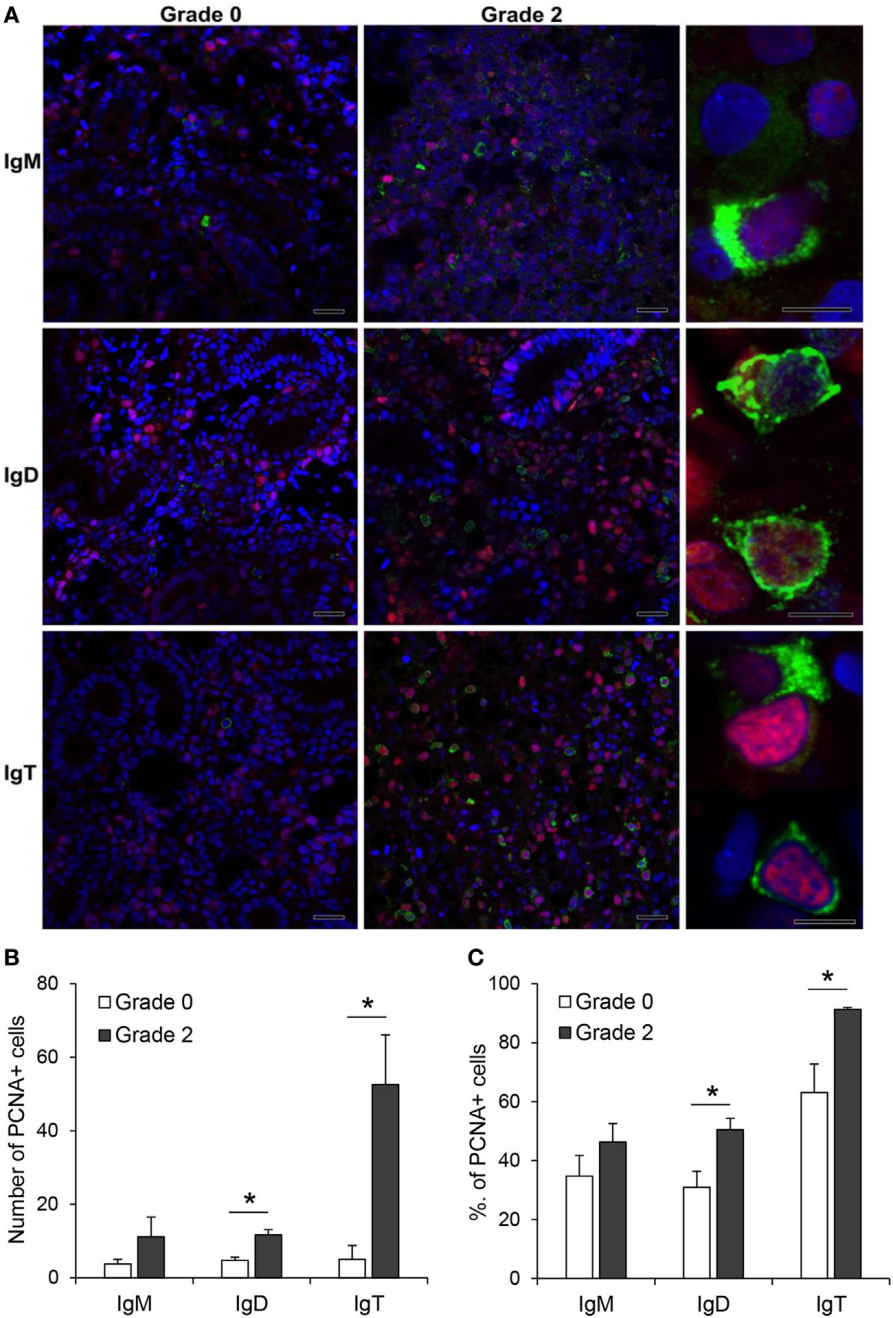
Proliferation of IgM^+^, IgD^+^, and IgT^+^ cells in kidney. **(A)** Confocal microscopic images of rainbow trout kidney sections infected with *Tetracapsuloides bryosalmonae* exhibiting different swelling grades were labeled with anti-immunoglobulin (Ig) M, anti-IgD, or anti-IgT (green) in combination with anti-proliferating cell nuclear antigen (PCNA) (red). All sections were also counterstained with DAPI (blue). Representative images for grade 0 and grade 2 are shown (scale bars = 20 µm) along with representative images from grade 2 kidneys at higher magnification (right; scale bars = 5 µm). Note that in non-proliferating cells, nuclei appear blue whereas they appear violet in proliferating cells. Mean number **(B)** and mean percentage **(C)** of proliferating IgM^+^, IgD^+^, and IgT^+^ cells were calculated in 10 digital fields (400× magnification) from 6 different individuals. Statistically significant differences (*P* < 0.05) in proliferating IgM^+^, IgD^+^, and IgT^+^ cells between grade 0 and grade 2 fish (analyzed by one-way ANOVA followed by two-tailed Student’s *t*-test) are indicated with an asterisk.

The increased expression of IgM, IgD, and IgT observed in the kidney of fish with clear signs of PKD pathology could be attributed to different factors such as increased differentiation of pre-B cells to mature Ig-expressing B cells, mobilization of B cells from other tissues or to local proliferation of B cells. Hence, to clarify this point, we performed double immunofluorescence staining with antibodies against the different Ig isotypes in combination with an antibody against PCNA, an intracellular molecule also linked to cell proliferation in fish, including salmonids (42–44). Through this methodology, we could verify that the number of IgM^+^, IgD^+^, and IgT^+^ B cells was higher in grade 2 kidneys than in grade 0 kidneys, as previously observed by immunohistochemistry. Nevertheless, the number of proliferating IgM^+^ B cells (PCNA^+^) was similar in grade 0 and grade 2 kidneys (Figure 6A,B), accounting for ~35 and ~46% of all IgM^+^ B cells, respectively (Figure 6C). By contrast, the number of proliferating IgD^+^ B cells (PCNA^+^) was higher in grade 2 kidneys than in grade 0 kidneys (Figure 6A,B), going from ~31 to ~50% of all IgD^+^ B cells (Figure 6C). Finally, the number of IgT^+^ proliferating B cells was dramatically increased in grade 2 kidneys when compared with kidneys with no visible signs of pathology (Figure 6B). In these tissues, almost all IgT^+^ B cells visualized in the preparations (~91%) were proliferating (Figure 6C).

### AID and TdT mRNA Levels in the Kidney Correlate With PKD Progression

As an initial approach to estimate how the Ig repertoire could be affected in response to *T. bryosalmonae*, we studied the levels of transcription of AID and TdT in kidneys affected by PKD at different stages of preclinical and clinical disease.

**Figure 7:**
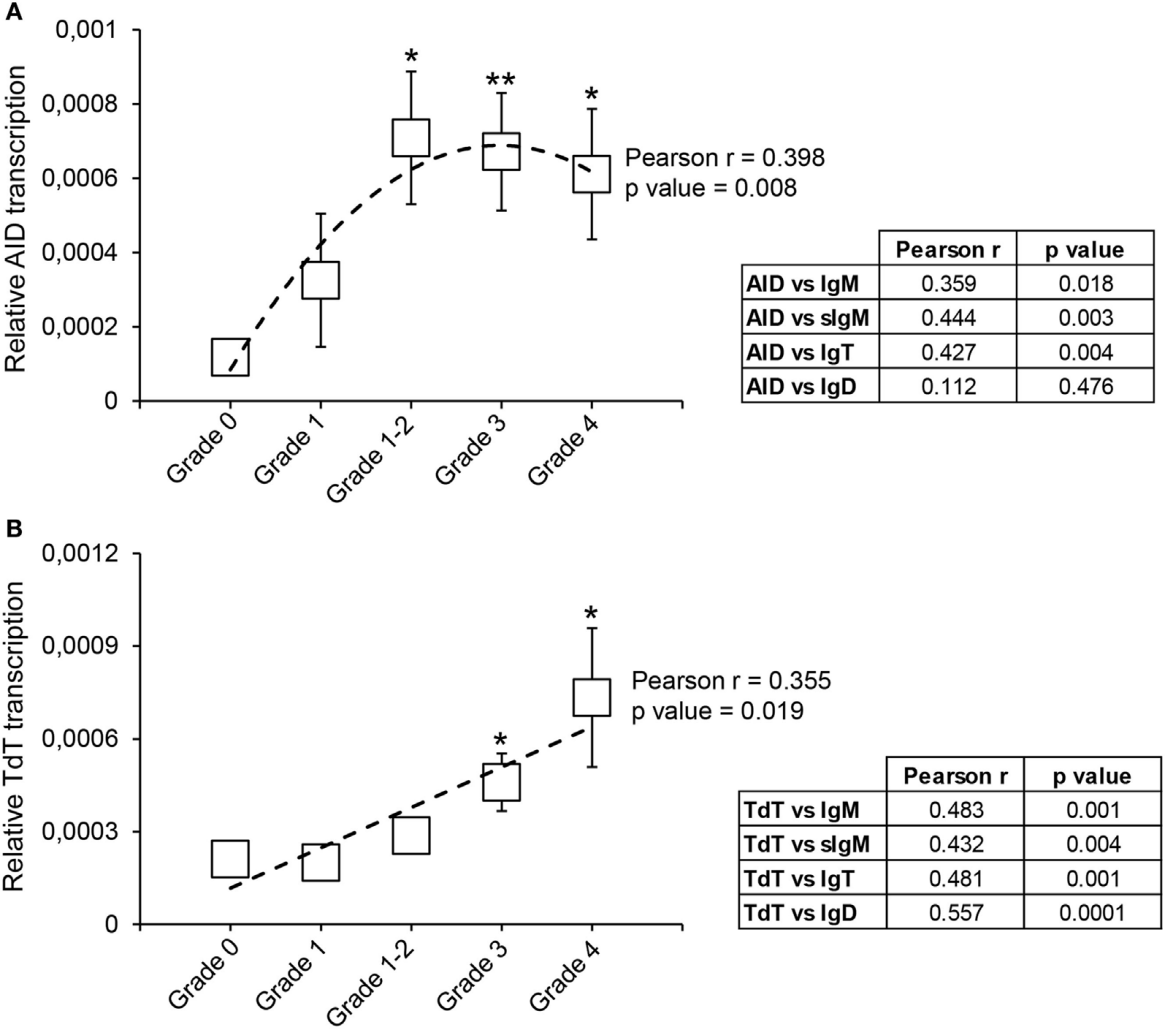
Transcriptional regulation of activation-induced deaminase (AID) and terminal deoxynucleotidyl transferase (TdT) during proliferative kidney disease. Transcriptional levels of AID **(A)** and TdT **(B)** were evaluated by real-time PCR in kidney samples classified according to their swelling grade. Results are shown as the gene expression relative to the expression of an endogenous control (EF-1α) (mean ± SEM) (G0, *n* = 10; G1, *n* = 4; G1–2, *n* = 7; G2, *n* = 11; and G3, *n* = 9). Statistical differences between control and infected groups (analyzed with a two-tailed Student’s *t*-test) are shown as *P < 0.05 and **P < 0.01. A polynomic or linear regression is also shown (dotted line) to reveal the correlation between the expression of specific genes and the progression of the pathology, together with the Pearson product-moment correlation coefficient (*r*) and the statistical significance of the correlation (*P* value), which are indicated in the plots. Pearson product-moment correlation coefficient (*r*) and statistical significance of the correlation (*P* value) between AID **(A)** or TdT **(B)** transcription with the transcription of immunoglobulin (Ig) M, secreted IgM (sIgM), IgT, and IgD are included in the adjacent tables (right).

Our results show that from grade 1–2 the levels of transcription of AID are significantly upregulated in comparison with the levels detected in grade 0 fish (Figure 7A). This difference is maintained in grade 3 and grade 4 fish, although with mRNA levels slightly lower than those at grade 1–2 (Figure 7A). Despite this, there was a significant correlation between the levels of AID transcription and the extent of clinical disease (Figure 7A). To gain some insights on which Ig could be affected by the action of AID, we also evaluated the correlation between AID mRNA levels and mRNA levels of the different Igs. As shown in Figure 7A, AID mRNA levels significantly correlated with total IgM, sIgM, and total IgT mRNA levels, but not with IgD mRNA levels (Figure 7A).

The transcription of TdT was also upregulated throughout the course of clinical disease, reaching mRNA levels, at grades 3 and 4, significantly higher than those found in fish with no visible signs of pathology (Figure 7B). Furthermore, a significant correlation between the levels of TdT transcription and the kidney swelling grade was clearly visible (Figure 7B). Interestingly, the levels of transcription of TdT significantly correlated with the levels of transcription of all Ig isoforms (Figure 7B).

### VH Family Usage During PKD Progression

Previous reports have analyzed in depth the repertoire of IgM, IgD, and IgT in trout exposed to a viral infection (21). Thus, we followed the protocol described in that study by Castro et al. to amplify heavy-chain rearranged transcripts (IGH V–D–J–C) using a set of isotype-specific primers for IgM, IgD, and IgT, respectively, and a set of IGHV subgroup-specific primers that can amplify all members of the 13 known IGHV groups (Figure S1 in Supplementary Material). We performed this analysis in four grade 0 fish and four fish with grade 2 pathology, given that it was at this stage that the levels of Ig protein expression peaked. All PCRs belonging to one fish were subsequently pooled and deep sequenced to obtain as much information as possible regarding the repertoire of the three Ig isotypes. Using Illumina MiSeq (2 × 300), an average of 2.4 million paired reads per sample were obtained (raw data). Total sequences were cataloged using reverse primers as barcodes for isotype identification. Afterward ~88, ~75, and ~90% of the sequences were classified as productive sequences after IMGT/HighV-QUEST analysis for IgM, IgD, and IgT, respectively (Table S3 in Supplementary Material). Within this set of sequences, IgM was the most common isotype, accounting for ~90% of the sequences, whereas IgD and IgT accounted for around 2–5% of sequences. The accuracy of the PCR and MiSeq sequencing was analyzed using a 50 bp fragment from the IgM constant region. A total of 757 Mbp from the eight samples was used to estimate an average error ratio, which was 4.01 × 10^−3^. This value was concordant with values observed previously (21) and was considered low enough to evaluate SHM in Ig genes.

Unique sequences defined as V(D)J rearrangements associated with a specific CDR3 amino acid sequence were grouped into what has been previously cataloged as junction sequence types (JST) (23). Globally, a significant increase in number of unique sequences was identified for IgT in comparing grade 0 and grade 2 fish, whereas no significant changes were observed for IgM and IgD (Figure S2 in Supplementary Material). However, significant changes were identified for all isotypes with respect to the VH family usage.

**Figure 8:**
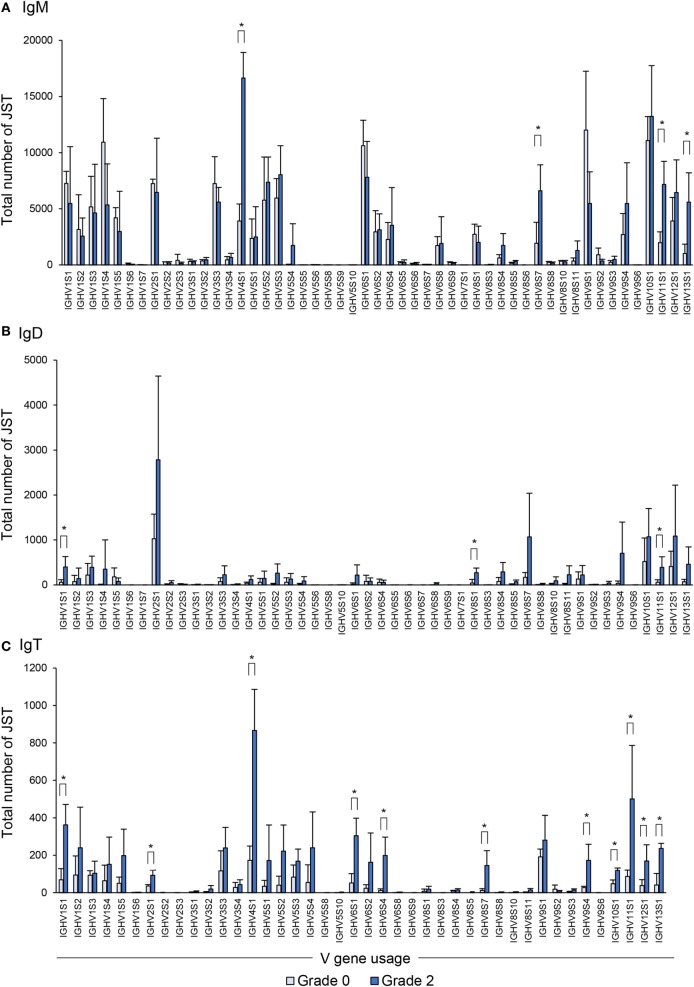
Differential usage of germline immunoglobulin (Ig) VH-gene segments during the B cell response to proliferative kidney disease. Bar charts show the average number (mean + SD) of unique sequences [V(D)J-CDR3(AA)] defined as junction sequence types (JST) identified for IgM **(A)**, IgD **(B)**, and IgT **(C)** for each VH family in grade 0 kidneys compared with grade 2 (*n* = 4). Statistical differences (*P* < 0.05) between the two groups (analyzed with a two-tailed Student’s *t*-test) are shown with an asterisk.

Concerning IgM, as reported before in clonal trout (21), the rainbow trout analyzed also used a broad range of VH families whereas only a few families were not used by any of the fish studied (Figure 8A). In comparing the VH family usage between grade 0 and grade 2 fish, we observed an increased use of some of these families (IGHV4S1, IGHV8S7, IGHV11S1, and IGHV13S1) (Figure 8A).

When the IgD repertoire was analyzed, we found that, in general, IgD used a lower number of VH families than IgM (Figure 8B). In this case, B cells expressing IgD associated with IGHV1S1, IGHV8S1, and IGHV11S1 were significantly overrepresented in the kidney of fish exhibiting grade 2 pathology in comparison to fish with no evident signs of pathology (Figure 8B), although the increases detected were modest when compared with those found for other isotypes. However, this different profile of IgD compared with IgM in VH family expansion strongly suggests a certain degree of differential selection.

Similar to the IgM results, B cells expressing IgT use a wide repertoire of VH groups (Figure 8C). However, when we compared the IgT repertoire of grade 0 and grade 2 kidneys, we found that up to 11 VH families were overrepresented in B cells found in grade 2 kidneys in comparison with those present in kidneys with no evident signs of pathology (Figure 8C). These were IGHV1S1, IGHV2S1, IGHV4S1, IGHV6S1, IGHV6S4, IGHV8S7, IGHV9S4, IGHV10S1, IGHV11S1, IGHV12S1, and IGHV13S1 (Figure 8C). Overall these results indicate that, throughout the course of PKD progression, B cells bearing Igs with specific VH segments are positively selected. As many different VH families are expanded, such results point to a polyclonal activation of B cells. This is especially evident in the case of IgT-expressing cells whereas it is less pronounced for IgM and IgD.

### V(D)J Recombination Repertoire

We also analyzed the V(D)J recombination repertoire in each of the fish studied and created heatmaps showing the relative occurrence of each V(D)J recombination within the repertoire of each Ig isotype. These heatmaps revealed a large number of trends that were apparent only when analyzing the repertoire in the context of complete V(D)J recombinations. For IgM, we found that the VH families that were previously seen overrepresented in grade 2 fish, were mostly increased in association with IGHJ3, IGHJ4, IGHJ5, and IGHJ6 families (Figure S3 in Supplementary Material). A similar response was observed for IgD (Figure S3 in Supplementary Material), while the VH families that were significantly expanded for IgT in grade 2 fish were always associated with IGHJ1 and IGHJ2 segments (Figure S3 in Supplementary Material). Interestingly, the fact that IgT only uses IGHJ1 and IGHJ2 segments was also reported in a previous study using clonal rainbow trout (21). This specific expansion of V–J pairs for the three Igs was not so evident when V–D associations were studied in the case of IgM and IgD, as the VH families expanded could associate with any D segment (Figure S4 in Supplementary Material). However, again, the IgT expanded families were preferentially associated with IGHD1, IGHD2, and IGHD3 (Figure S4 in Supplementary Material).

### JST Analysis During PKD Progression

**Figure 9:**
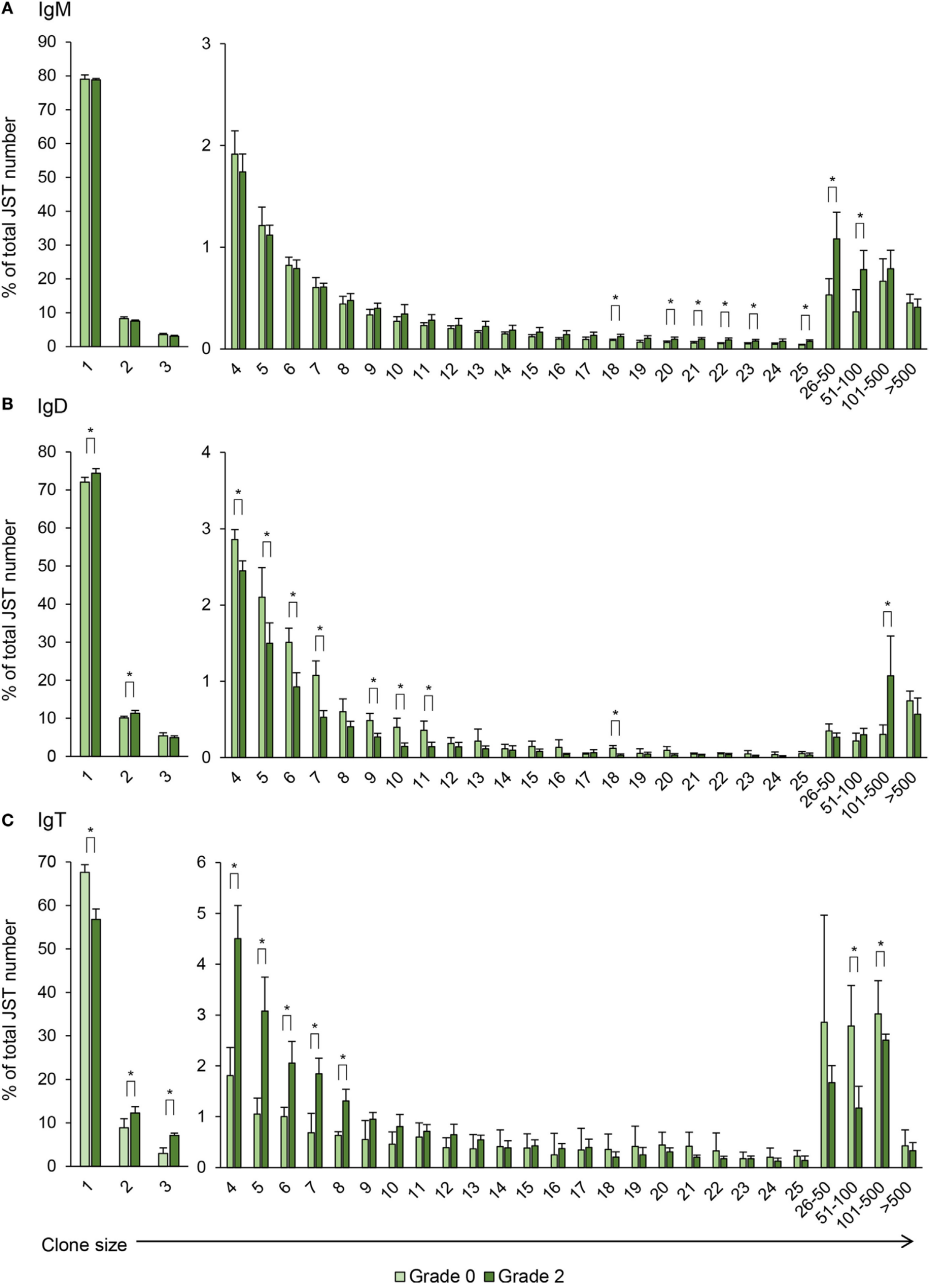
Clonal size distribution of junction sequence types (JST) during B cell response to proliferative kidney disease. Bar charts show the average percentage (mean + SD) of JST observed *n* times in the sequence datasets for IgM **(A)**, IgD **(B)**, and IgT **(C)** from grade 0 kidneys in comparison with grade 2 kidneys (*n* = 4). Statistical differences (*P* < 0.05) between the two groups (analyzed with a two-tailed Student’s *t*-test) are shown with an asterisk.

Previous studies have established that JST which are found less than three to five times correspond to naïve non-expanded B cells whereas JST found more than 50 times correspond to highly expanded clones, possibly antibody-secreting cells (23). For IgM, around 80% of the JST were found only once in fish with no clinical signs indicating a broad diversity of the IgM repertoire in the kidney of these animals (Figure 9A). However, the JST profile obtained for grade 0 kidney samples was quite different from that previously described for rainbow trout spleen. In spleen, very few JST were found more than 20 times, in concordance to the presence of only a few antibody-secreting cells in a healthy spleen (21). In our studies, there was a considerable number of JST found more than 20 times, with some represented more than 100 times. This possibly reflects the presence of plasmablasts and plasma cells previously reported in the kidney of unstimulated trout (4). Interestingly, as PKD progressed, the number of JST represented 16–100 times was significantly increased (Figure 9A), again pointing to an oligoclonal expansion of IgM^+^ B cells.

For IgD, around 70% of the JST were found only once in fish with no clinical signs of disease and interestingly this percentage increased in grade 2 kidneys, as well as JST found twice (Figure 9B). On the other hand, the percentages of JST found in relatively small numbers (from 4 to 11 times) were significantly decreased in grade 2 kidneys in comparison with grade 0 kidneys, whereas the percentage of JST with large copy numbers (from 101 to 500) significantly increased for IgD (Figure 9B). Again, the differences in the JST profile in response to PKD pathogenesis between IgM and IgD strongly suggest a differential regulation of both Igs.

Similar to IgM, the JST distribution for IgT in fish with no clinical signs of disease pointed to the presence of some expanded clones, possibly IgT-secreting plasmablasts or plasma cells already present in these kidneys (Figure 9C). Despite this, in these fish, around 70% of the JST were only represented once, indicating a high degree of repertoire diversity (Figure 9C). However, with increasing clinical disease, the percentage of JST that were only represented once was significantly reduced to ~60% whereas the number of JST represented from two to eight times was significantly increased (Figure 9C). Interestingly, the IgT JST represented more than 100 times was reduced in grade 2 fish (Figure 9C). Thus, overall, our results seem to indicate that advanced clinical disease induces the expansion of a large number of IgT clones that became activated but do not fully differentiate into plasma cells.

### CDR3 Spectratyping of Igs During PKD Progression

Taking into account that the CDR are the regions where BCR bind to their specific antigen and that the CDR3 region of the VH gene is the most hyper-variable region of the BCR genes (45), the determination of CDR3 size by spectratyping has become a powerful tool to analyze the BCR cell repertoire under normal and pathological conditions in mammals and fish (21). Given that B cell clones differ in CDR3 length, the CDR3 length distribution analysis is an estimate of the overall diversity, and any deviation from a bell-shaped Gaussian distribution is indicative of clonal expansions. These clonal expansions can be monoclonal or oligoclonal depending on whether there is a single or several expanded peak(s) (46). In this study, we analyzed the CDR3 length distribution of unique sequences for IgM, IgD, and IgT using either total sequences or sequences that corresponded to the most expanded VH families, but no significant perturbations common to all grade 2 fish were found when compared with the profiles obtained in grade 0 fish in any case (Figure S5 in Supplementary Material). However, when these analyses were performed with total numbers of JST, they also revealed the expansion of B cell subsets with different CDR3 lengths, especially in the case of IgD and IgT (Figure S5 in Supplementary Material). These results suggest that the response to PKD does not involve the clonal selection of a specific B cell subset, but rather that it involves a poly/oligoclonal activation of different B cell subsets. Interestingly, it is worth noting that IgT apparently uses larger CDR3 segments than IgD and IgM, as previously reported by other authors (17, 21) and longer CDR3 have often been associated with autoimmunity and polyreactivity (47).

### Ig Mutation Rate During PKD Progression

**Figure 10:**
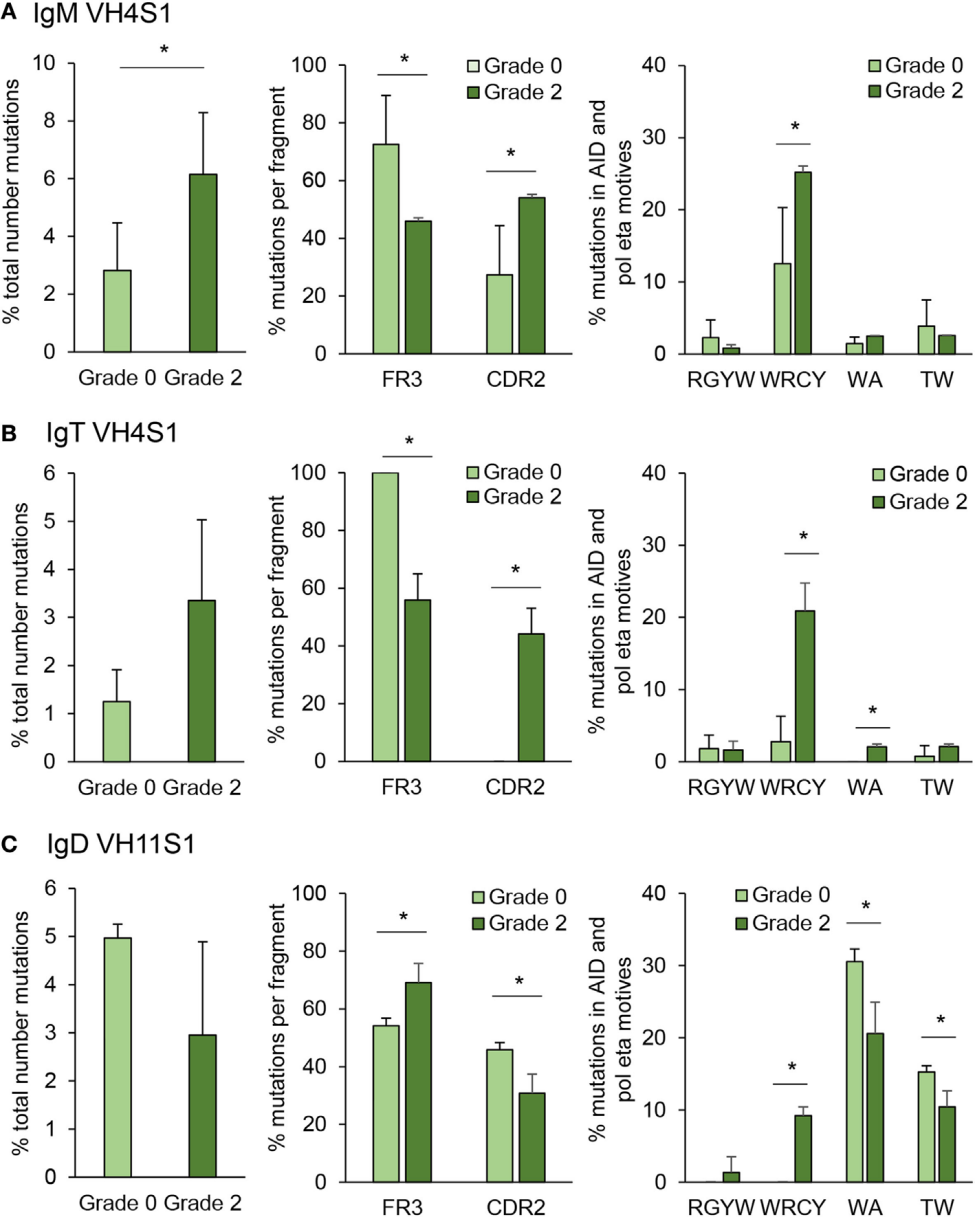
Targeting and patterns of somatic hypermutation identified for IgM **(A)**, IgT **(B)**, and IgD **(C)**. Bar charts show the average percentages (mean + SD) of mutations in grade 0 or grade 2 kidneys (*n* = 4), assessed by analyzing CDR2-FR3 region transcripts encoding the VH4 family from IgM and IgT or VH11 for IgD. Statistical differences (*P* < 0.05) between the two groups (analyzed with a two-tailed Student’s *t*-test) are shown with an asterisk.

Somatic hypermutation is a process driven by AID inside the GC to generate affinity maturation of antigen-selected GC B cells (48). Although fish do not contain GCs, they are known to express AID and have been reported to be capable of undertaking affinity maturation to a certain degree (27). However, whether SHM takes place extrafollicularly in fish or whether fish have primitive GCs is not yet defined and still under debate (49). In this study, we have quantified the mutation rate for IgM and IgT in IGHV4S1, a VH family preferentially expanded for both Igs and for IGHV11S1 in the case of IgD, as this was one of the VH families significantly expanded for IgD in response to PKD progression (Figure 8). In IgM, the percentage of total point mutations was significantly increased in grade 2 kidneys when compared with that observed in grade 0 kidneys (Figure 10A). Although the percentage of total point mutations also increased strongly in IgT, the differences were not significant due to a large variability among individuals (Figure 10B). Interestingly, in both cases, the mutations were accumulated in the CDR2 region as the disease progressed, with significant differences for both IgM and IgT, while they significantly decreased in the FR3 region (Figures 10A,B). Remarkably, in the case of IgT, 100% of the point mutations generated in the CDR2 in grade 2 kidneys were productive mutations (that implied a change in amino acid), whereas only a minor percentage of those accumulated in the FR3 region were productive (data not shown). For both IgM and IgT, most of these mutations were produced within the WRCY AID hotspot motif (Figures 10A,B), and mutations targeting this motif increased throughout PKD progression, possibly in concordance with increased mRNA AID expression (Figures 10A,B). No significant changes were observed, however, for the percentage of mutations within the RGYW motif, another AID hotspot. Mutations in A/T bases can also occur through the generation of mismatches introduced by error-prone polymerases such as polymerase (pol)η that introduces errors specifically in WA/TA motives (50). Although the percentage of mutations in these sites was much lower than those introduced at the WRCY AID hotspot, the mutations introduced at the WA motif were significantly higher in grade 2 fish than in fish with no signs of pathology in the case of IgT (Figures 10A,B). A completely different SHM profile was obtained for IgD. In this case, although the percentage of total mutations was not significantly increased in grade 2 kidneys when compared with grade 0 kidneys, the percentage of mutations within the FR3 regions significantly increased along with a significant decrease in the mutations within the CDR2 region (Figure 10C). Similarly, in human IgD^+^IgM^−^ B cells, the replacement mutations found in IgD in these cells were not concentrated within the CDRs (51). In this case, even though the higher mutation frequencies were observed with the WA/TA motives targeted by the (pol)η polymerase, their frequencies were reduced in response to PKD progression (Figure 10C), whereas those found with the WRCY AID hotspot significantly increased (Figure 10C), suggesting an involvement of AID in IgD SHM despite the fact that AID mRNA levels did not correlate with those of IgD (Figure 7).

## Discussion

A hallmark of adaptive immunity mediated by antibodies is the ability of B cells to generate immunological memory through which B cells can respond more rapidly and robustly producing specific antibodies upon re-exposure to pathogens. However, many parasites have developed strategies to manipulate the B cell response and escape adaptive immunity (52). Hence, although specific antibodies have been demonstrated to provide protection against some protozoan infections in mammals, it has also been shown that the B cell responses elicited by some of these parasites can be responsible for the induced pathogenesis. For example, *Trypanosoma cruzi, Neospora caninum*, and *Trypanosoma brucei* have the capacity to deplete B cell precursors in the bone marrow, thereby limiting the number of B cells in the periphery (53–55). By contrast, the spleen of individuals affected by many of these protozoan infections shows a marked cellular hyperplasia as a consequence of an intense B-cell response. Thus, parasites such as *T. cruzi* (56) or *Plasmodium* spp. (57) elicit strong extrafollicular B cell responses that lead to a strong hypergammaglobulinemia. Interestingly, while the total amount of antibodies markedly increases at early stages of infection with these parasites, the specific antibody titers are quite limited (56). Therefore, this polyclonal activation is a strategy used by some parasites to escape the host-specific immune response by means of diluting pathogen-specific antibodies while increasing irrelevant antibodies.

Previous transcriptional studies conducted in rainbow trout affected by PKD demonstrated an increased transcription of IgM and IgT that strongly correlated with the pathological score (9). Hence, in this study, we examined the production of IgM, IgD, and IgT at the protein level at different stages of clinical disease by both immunohistochemistry/immunofluorescence and Western blot analysis. Our results confirm a strong induction of Ig production in response to *T. bryosalmonae* in the kidney of infected fish. In the teleost fish, the kidney functions as both a hematopoietic tissue and as a secondary immune organ (4). Thus, while in the anterior kidney B cells develop and most proliferating B cell precursors are found, the posterior kidney houses significant populations of partially activated B cells and plasmablasts (4). This increased response, confirmed by both immunohistochemistry and Western blot, affected all Ig isotypes, namely, IgM, IgD, and IgT and was more robust in fish kidneys with grade 1–2 clinical swelling than in kidneys in which the disease had progressed to level 3–4. It should be noted that as all fish used in this study were sampled at one time point and fish with swelling grades from 1 to 4 were exposed to the parasite for the same period of time, the differences in swelling grades observed among individual fish might also be a result of different host responsiveness to the parasite. In any case, the repertoire analysis for all three Igs suggested that this increased expression of all three Igs is a result of a oligo/polyclonal B cell activation which might be an escape mechanism triggered by *T. bryosalmonae*, being also, at least in part, responsible for the pathogenesis associated with the parasite proliferation. These results seem in correlation with the serum IgM hyperimmunoglobulinemia that is induced by PKD infection (58).

Previous transcriptional studies had reported significant upregulation of IgM and IgT mRNA levels with increasing clinical disease, but not of IgD mRNA levels (9). However, an evident upregulation of IgD protein has been demonstrated in this study. Previous studies performed in mice have established that the basal level of IgD transcription remained constant in different B cell subsets regardless of their levels of IgD surface expression (59), leading to the hypothesis that posttranscriptional processing plays an important role in the changes in expression of IgD during B cell differentiation. Thus, it could be possible that posttranscriptional regulation also conditions the amount of IgD produced by different B cell subsets in teleosts. To date, the precise role of IgD in the immune response is still largely unknown in mammals and fish (14). Although generally attributed a minor immune role due to the fact that IgD-deficient mice are able to mount normal T-dependent and T-independent immune responses (60), the identification of IgD-secreting cells in specific mucosal surfaces in mammals and the fact that it has been conserved throughout evolution points to a relevant but still unknown role (14). In this study, we provide strong evidence of a specific regulation of IgD in response to antigenic stimulation at the protein level, as well as a clonal expansion of IgD-expressing B cells in response to PKD progression for the first time in teleost fish. Since we have demonstrated the presence of IgD^+^IgM^−^ cells in parasite-exposed kidneys, it seems probable that these are the cells clonally expanded in response to the parasite. In mammals, despite the absence of a repetitive S region upstream of C_δ_, a non-canonical CSR process that is still not fully understood generates cells that exclusively produce IgD after an AID-mediated deletion of C_µ_ (51, 61). Whether teleost IgD^+^IgM^−^ cells also appear as a consequence of a non-canonical CSR is something that should be further explored. Interestingly, although the SHM profile of IgD was quite different to that of IgM and IgT, a certain degree of AID-mediated SHM was found associated with PKD disease progression. In humans, IgD^+^IgM^−^ B cells show a very high rate of SHM (51), whereas equivalent population in mice show a very low mutation rate (62). Thus, the identification of IgD^+^IgM^−^ cells in the kidneys of PKD-infected fish, as well as the demonstration of SHM and clonal expansion of IgD throughout the progression of the disease, challenges the hypothesis that IgD functions simply as an antigen receptor and adds to previous evidence in mammals that points to an important role of IgD in specific humoral responses (51, 63).

Despite the regulation of IgM and IgD throughout the course of PKD, our results unequivocally point to a prevailing role of IgT in the immune response to the parasite given its dominant role in parasite coating, B cell proliferation, clonal expansion, and SHM. Interestingly, already in 2002, Chilmonczyk et al. demonstrated that the lymphocytes that were proliferating in response to PKD were mostly IgM^−^ (8). Even though it could be possible that some T cells are also proliferating in response to the parasite, their results are in concordance to the preferential proliferation of IgT^+^ B cells in PKD. The fact that IgT is the main Ig isotype that is modulated in the kidney during the progression of PKD seems surprising given that IgT has been reported to be as a specialized mucosal Ig (18–20); however, previous reports have also demonstrated non-mucosal roles for IgT (18, 21, 22). Interestingly, in all the studies that pointed to a mucosal role of IgT a parasite model was used (18–20), therefore, it also seems plausible that IgT plays a leading role in the immune response to myxozoan/protozoan parasitic infections, given the absence of IgE in teleosts. Previous studies support this idea, since IgT was the only Ig isotype induced in the kidney upon infection with a myxozoan parasite in gilthead sea bream (*Sparus aurata*) (64). Our study also revealed several aspects of IgT not previously explored in fish. For example, we have seen that similar to IgM, expanded IgT JST sequences are present in the rainbow trout kidney, suggesting the presence of IgT plasmablasts/plasma cells in animals with no evident signs of disease. Thus, whether the kidney constitutes a survival niche for IgT plasmablasts/plasma cells as described for IgM plasma cells (4) is something worth exploring further. In addition, we have confirmed for the first time SHM for IgT, demonstrating that through the course of PKD, mutations could accumulate for IgT in the AID hotspot WRCY. The fact that AID is able to mutate fish Igs in the absence of recognizable GCs also merits additional investigation in the future.

In conclusion, we provide novel results that substantiate the upregulation of all three Ig types in response to PKD at the protein level, not seen previously by transcript studies (e.g., in regards to IgD). Additional repertoire analysis further demonstrates that the upregulation is a consequence of a polyclonal expansion of the different Ig subsets found in the kidney during PKD, given that no public or private monoclonal responses similar to those previously reported in response to viral infection (21) were identified. This study confirms an important role of IgD in the humoral response to the parasite, which includes the appearance of IgD^+^IgM^−^ B cells, SHM, and clonal expansion of some IgD-expressing B cell subsets. Nonetheless, in addition to the regulated IgM and IgD responses, IgT was also regulated in the kidney in response to the parasite, playing a prevailing role that confirms the fact that IgT is an important player outside of mucosal compartments. Our results provide important novel data to further understand the regulation of Ig synthesis in teleosts and delivers valuable information to aid future intervention strategies against PKD.

## Ethics Statement

This study was approved by Instituto Nacional de Investigación Agraria y Alimentaria (INIA) Ethics Committee (ORCEEA 2016-021).

## Author Contributions

BA performed the immunohistochemical analysis of Ig production, Western blots and real-time PCR analyses. IE determined the parasite coating and performed all immunofluorescence assays and analyses. PP undertook all the analyses related to the Ig repertoires. MF and YH performed the fish sampling and produced cDNAs and protein extracts. CT and JH designed the experiments. CT wrote the main body of the paper with contributions from JH, AG, PR, and CS.

## Conflict of Interest Statement

The authors declare that the research was conducted in the absence of any commercial or financial relationships that could be construed as a potential conflict of interest.
