# Supplementary material for: Dysregulation of B Cell Activity During Proliferative Kidney Disease in Rainbow Trout
Source: Front Immunol. 2018 May 31;9:1203. doi: 10.3389/fimmu.2018.01203 (PMC5990594; doi:10.3389/fimmu.2018.01203)
Supplement: Supplementary file 1 [file Presentation_1.PDF]

Table S1. Primers used in this study for real time analysis

Gene	Primer name	Primer sequence (5´-3´)
IgMtotal	RT-IgMtotal-F	TGCGTGTTTGAGAACAAAGC
	RT-IgMtotal-R	GACGGCTCGATGATCGTAAT
IgMsec	RT-IgMsec-F	CCTTAACCAGCCGAAAGGG
	RT-IgMsec-R	TGAGGTTCTATCAATGGTTCTC
IgT	RT-IgTtotal-F	AACATCACCTGGCACATCAA
	RT-IgTtotal-R	TTCAGGTTGCCCTTTGATTC
IgD	RT-IgDtotal-F	AGCTACATGGGAGTCAGTCAACT
	RT-IgDtotal-R	CTTCGATCCTACCTCCAGTTCCT
AID	RT-AID-F	TCCGCCTCAGGATCTACGTCTC
	RT-AID-R	GCCAACAGTAGAAATAGTCTTCATAGTTCATG
TdT	RT-TdT-F	AGAGGTCAAGTTCAAGGAGGTGACT
	RT-TdT-R	ACTGAGGACATCTTCCACCCTAAAG
EF-1α	RT-EF1α-F	GATCCAGAAGGAGGTCACCA
	RT-EF1α-R	TTACGTTCGACCTTCCATCC

Table S2. Primers used in this study for repertoire analysis

Primer name	Primer sequence	IMGT name of IgV genes
VH1.1-F	AGCAGTGATGGTGGCAGCACT	IGHV1S1; Sc2184VH3r
VH1.2-F	CAATTAGTGATTCAAGCAGTTATA	IGHV1S2
VH1.3-F	CAGCGCATTATGACATTAGAAATA	IGHV1S3;Sc68806VHr
VH1.4-F	TTCTGCACCAAGTGGAGCTGACAA	IGHV1S5
VH1.5-F	TATGATAGTGCTGAAATCTACTAC	IGHV1S4; IGHV1S6; IGHV1S7 ; Sc5246VH1
VH2-F	GGTCTGAGAGCAGAGGACTCTGC	IGHV2S1 to IGHV2S3 ; Sc24807VH , Sc2184VH6r, Sc4731VH6, Sc4731VH7, Sc2931VH2, Sc30311VH, Sc71265VH1, Sc79393VH
VH3.1-F	CATGTGTGGCAGTGGTAACATA	IGHV3S1 to IGHV3S4; Sc5246VH2
VH3.2-F	TTGTTACAATGGTAACACATATG	Sc4000VH
VH4-F	ACTCTGGTTCAACAGATGCTCCAGTC	IGHV4S1
VH4.2-F	TGGATTGGAAGAATGAACACTGG	Sc33539VHr
VH5.1-F	TACACACTGGTGGATCGAGTC	IGHV5S2; IGHV5S5; IGHV5S6; IGHV5S7; IGHV5S8; IGHV5S9; Sc5246VH3
VH5.2-F	CAGCACAGCTAGTACACCCAT	IGHV5S3; Sc20487VH
VH5.3-F	TGGATTGC(C/T)TATAGTTATAGTAC	IGHV5S4; Sc2184VH1r; Sc2931VH1; Sc54572VH
VH5.4-F	ATATTAGCACACAGAGTAATCC	IGHV5S1
VH6-F	CTGAAAAA(C/T)AAGTTCAGCCTC	IGHV6S1 to IGHV6S9 ; Sc4731VH1 ; Sc7987VHr; Sc934VHr; Sc11506VH; Sc4731VH4; Sc4731VH5
VH7-F	GTTCAGCATTTCAACACATGC	IGHV7S1
VH7.2-F	CAAGTTCAGCTTTAGAAGAGAC	Sc4731VH3
VH8-F	AAGGACAGCA(G/C)(A/T)AATTTCTAT CTG	IGHV8S1 to IGHV8S11 ; Sc9908
VH9.1-F	CTGGAACCACTGCTTATTATGC	IGHV9S3 ; IGHV9S4; IGHV9S5; IGHV9S6; Sc2931VH4r
VH9.2-F	TGGCACTGGCACTGTATTTGC	IGHV9S1; IGHV9S2; Sc37982VH1 ; Sc15217VH ; Sc2184VH4r ; Sc2931VH0
VH10-F	AGCAAGATCACTTCTAGATATG	IGHV10S1
VH11.1-F	TGGAGTGGATTGGGATCATCTG	IGHV11S1; Sc4731VH8; Sc7987VH2
VH12-F	GGACCATCTATTATGATGGAAGC	IGHV12S1
VH13-F	GATACCTGTGTAGTAGTAGTAGC	IGHV13S1; Sc2184VH5r
IgCμ2-R	AGAGACGGCTGCTGCAGATATTCC	
IgCδ4-R	TGGACTGGAGATGTGGTCAC	
IgCtau2-R	GATGTCGTTAGAAGGGGTTCCA	

Fig. S1

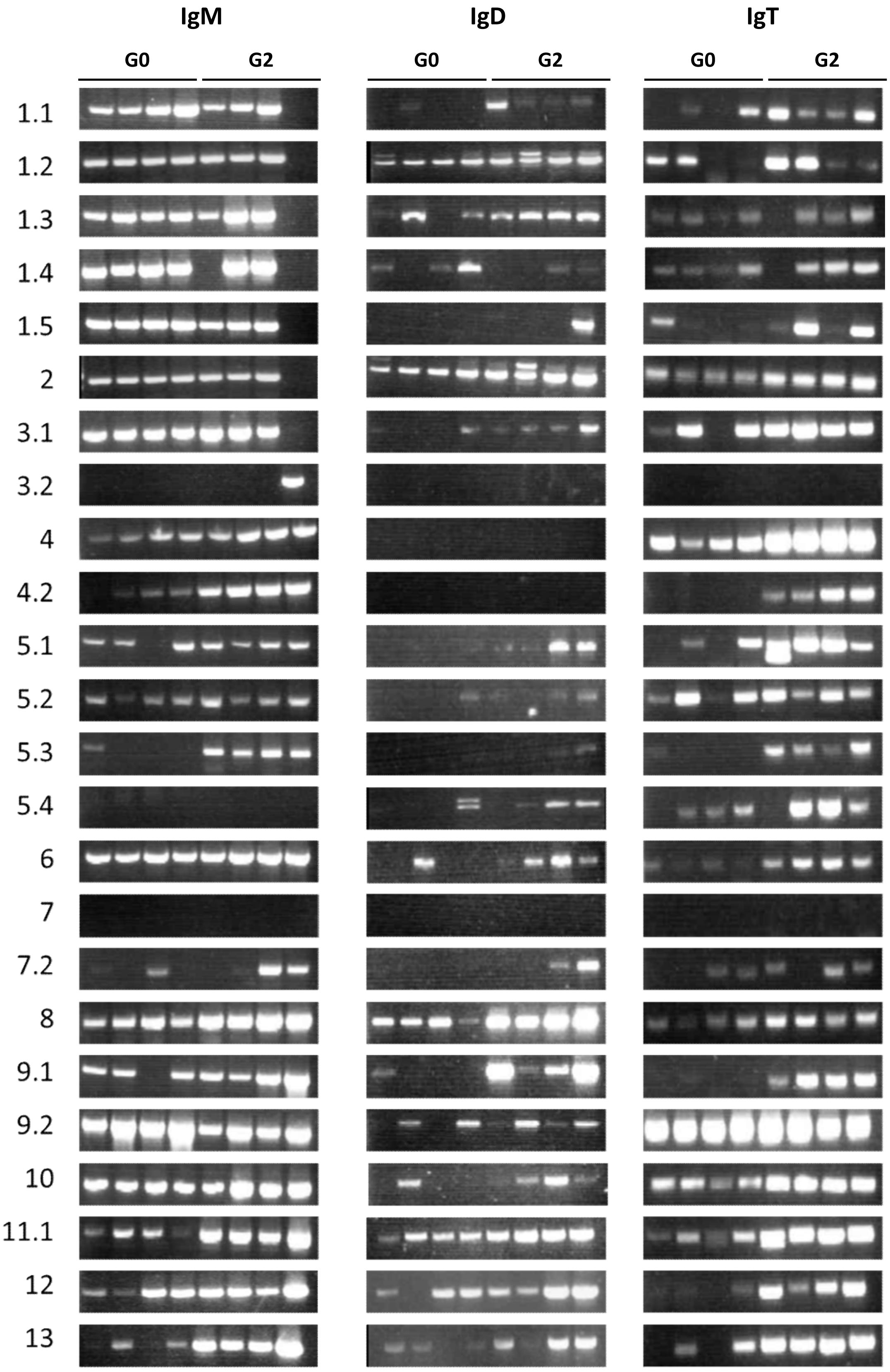

Figure S1. Amplification by PCR of the different rearranged VH-Cμ , VH-Cδ and VH-Cτ combinations of the BCR in 4 individual Grade 0 kidneys and 4 showing Grade 2 pathology. PCR was performed combining a VH family specific forward primer with a C specific reverse primer. To undertake Illumina MiSeq sequencing, 2 μl of all PCR products belonging to one individual were pooled together.

Table S3. Summary of sequence analysis. The total sequences column represents previously catalogued sequences using reverse primers as barcodes for isotype identification. These correspond to the initial datasets for IMGT/HighV-QUEST analysis. IMGT results were catalogued in productive or unproductive sequences using the immune repertoire pipeline from Antigen Receptor Galaxy (ARGalaxy).

	Sample	Total Sequences	Productive	%	Unique Productive	%	Unproductive	%	Unique Unproductive	%
IgM	S1	2,003,616	1,794,212	89.55	120,448	6.01	173,180	8.64	102,615	5.12
	S2	2,008,223	1,790,672	89.17	147,569	7.35	178,869	8.91	121,092	6.03
	S3	1,721,711	1,521,980	88.4	108,348	6.29	165,454	9.61	96,164	5.59
	S4	1,813,343	1,610,689	88.82	118,116	6.51	171,134	9.44	97,428	5.37
	S5	1,669,168	1,483,980	88.91	112,342	6.73	155,623	9.32	91,204	5.46
	S6	2,096,373	1,868,928	89.15	148,552	7.09	186,608	8.9	123,357	5.88
	S7	2,166,026	1,843,681	85.12	172,122	7.95	272,390	12.6	143,919	6.64
	S8	2,007,524	1,749,147	87.13	146,348	7.29	222,305	11.1	126,360	6.29
IgD	S1	36,053	28,576	79.26	2,166	6.01	5,130	14.2	1,310	3.63
	S2	86,490	71,328	82.47	5,117	5.92	10,186	11.8	2,757	3.19
	S3	62,625	48,496	77.44	3,662	5.85	10,188	16.3	1,808	2.89
	S4	63,629	43,912	69.01	3,396	5.34	14,529	22.8	2,758	4.33
	S5	116,240	89,920	77.36	8,371	7.2	19,894	17.1	4,828	4.15
	S6	77,314	52,023	67.29	4,812	6.22	9,770	12.6	2,,608	3.37
	S7	139,415	97,328	69.81	10,600	7.6	34,794	25	7,278	5.22
	S8	262,669	205,394	78.19	22,429	8.54	44,916	17.1	12,494	4.76
IgT	S1	40,839	38,881	95.21	1,799	4.41	1,570	3.84	507	1.24
	S2	31,092	29,417	94.61	1,544	4.97	1,442	4.64	419	1.35
	S3	11,915	9,810	82.33	683	5.73	559	4.69	155	1.3
	S4	29,033	26,119	89.96	1,930	6.65	1,690	5.82	560	1.93
	S5	63,159	58,178	92.11	3,432	5.43	3,572	5.66	1,040	1.65
	S6	52,388	49,405	94.31	6,658	12.7	2,084	3.98	1,095	2.09
	S7	59,964	52,220	87.09	5,997	10	7,345	12.2	1,658	2.76
	S8	66,611	57,406	86.18	5,891	8.84	7,642	11.5	1,751	2.63

Fig. S2

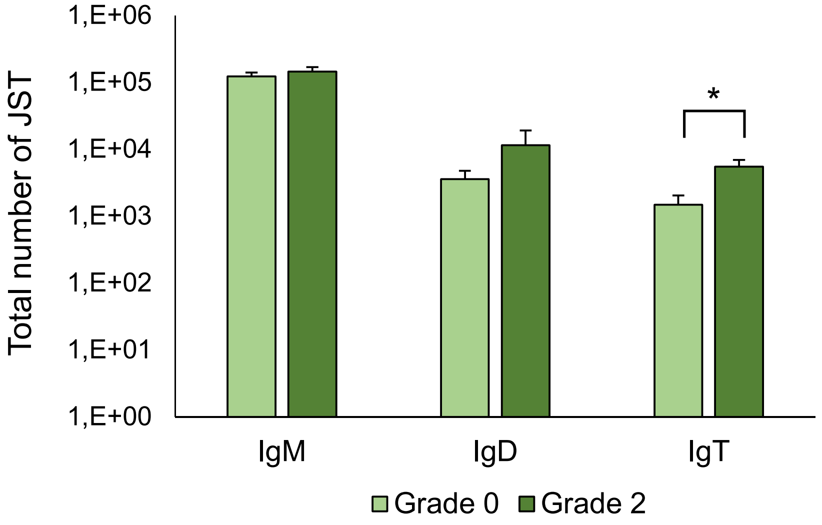

Figure S2. Total number of JST identified in IgM, IgD and IgT. Bar charts show the mean (+SD) number of total JST identified for each isotype in Grade 0 kidneys in comparison to Grade 2 kidneys (n=4). Statistical differences ($P<0.05$) between control and infected groups are indicated by an asterisk.

Fig. S3

V-J

IgM

	GRADE 0							GRADE 2						
	IGHJ1	IGHJ2	IGHJ3	IGHJ4	IGHJ5	IGHJ6	IGHJ7	IGHJ1	IGHJ2	IGHJ3	IGHJ4	IGHJ5	IGHJ6	IGHJ7
**IGHV1S1**	7,0	2,3	592,3	2487,3	2688,5	1354,8	124,0	4,8	3,0	526,8	1630,8	2327,3	810,8	168,3
**IGHV1S4**	6,8	4,5	1282,5	4377,3	2994,0	1936,5	333,0	5,5	4,3	321,3	1553,0	1993,5	1362,3	107,0
**IGHV1S5**	5,3	1,8	633,5	1041,3	1195,0	1239,0	79,3	7,0	1,0	435,5	626,8	474,8	1356,8	83,0
**IGHV2S1**	1,7	2,3	1006,0	2016,5	2844,0	1198,0	183,0	**5,3**	1,0	811,0	1711,3	2399,5	1303,3	229,8
**IGHV4S1**	3,0	3,0	539,3	1392,0	1095,0	705,5	176,5	**21,5**	**9,8**	**2601,8**	**3728,5**	**5981,8**	**3776,0**	519,8
**IGHV5S2**	10,0	7,3	325,0	1844,5	2875,5	696,3	12,8	6,0	5,5	565,8	2340,3	3089,3	1319,8	43,3
**IGHV6S1**	8,5	4,0	835,0	3075,3	4323,0	2153,5	215,0	9,8	4,0	830,5	2121,8	3274,0	1463,5	114,5
**IGHV6S4**	1,5	1,7	253,8	629,5	766,5	578,0	32,5	2,0		332,3	790,5	1176,0	1122,5	106,3
**IGHV8S1**	4,0	1,0	109,8	868,3	1096,8	539,0	118,5	1,8	**2,3**	148,8	679,0	632,5	517,0	39,8
**IGHV8S4**	1,0	1,0	84,5	231,3	197,8	99,8	16,3	1,7	1,5	101,8	779,0	**554,3**	276,8	31,3
**IGHV8S7**	2,5	3,0	275,3	713,0	665,3	215,3	53,3	4,0	2,5	481,8	2615,3	**2558,8**	**857,5**	93,3
**IGHV8S11**	1,7	1,0	38,0	143,3	126,5	44,0	8,0	1,0	1,0	69,5	585,8	**402,3**	210,3	25,5
**IGHV9S1**	9,0	6,3	1061,5	3391,5	5392,0	1768,8	379,5	3,3	1,7	645,5	2223,8	1497,5	1013,0	87,8
**IGHV9S4**	2,5	2,0	254,8	598,8	1143,3	532,3	183,0	3,5	3,8	323,5	**1899,5**	1938,3	1110,8	191,0
**IGHV10S1**	15,0	6,3	1382,5	4176,3	3509,8	1460,3	523,3	21,3	7,3	2148,0	3734,0	3109,0	**3976,5**	**234,8**
**IGHV11S1**	3,8	3,0	108,8	478,8	1069,0	265,0	60,5	9,5	9,5	384,5	2002,0	**2903,0**	**1490,8**	370,8
**IGHV12S1**	2,7	3,3	368,8	1362,3	1387,8	546,3	248,5	7,3	4,8	1135,5	1291,0	2456,0	1379,5	170,5
**IGHV13S1**	2,5	4,0	105,5	370,0	272,5	195,3	69,0	8,5	3,3	472,5	**1076,0**	2343,3	**1618,8**	74,3

IgD

	GRADE 0							GRADE 2						
	IGHJ1	IGHJ2	IGHJ3	IGHJ4	IGHJ5	IGHJ6	IGHJ7	IGHJ1	IGHJ2	IGHJ3	IGHJ4	IGHJ5	IGHJ6	IGHJ7
**IGHV1S1**			22,3	8,0	14,5	22,0		1,0		32,3	**125,0**	**164,0**	73,3	7,3
**IGHV1S4**			5,5	1,8	2,3	2,5	1,0			24,3	88,3	149,5	113,0	16,0
**IGHV1S5**			73,5	33,3	51,0	80,5				2,5	32,3	2,0	52,0	12,5
**IGHV2S1**		1,0	177,0	305,0	313,5	228,5	2,0	2,7	1,5	373,3	863,3	1004,5	464,0	100,0
**IGHV4S1**			5,7	14,0	6,8	14,8	1,0	1,0		21,0	33,0	**45,0**	15,5	4,8
**IGHV5S2**	1,0		1,0	3,7	14,3	1,0		1,0		31,0	57,8	135,5	81,7	
**IGHV6S1**	1,0			2,0	57,0	1,7	1,0	1,0		22,0	79,3	102,8	10,8	20,0
**IGHV6S4**			8,3	44,3	2,3	3,0				7,0	16,3	18,3	21,5	1,7
**IGHV8S1**			54,3	1,3	1,0	3,0			1,0	14,0	65,8	113,5	76,5	2,0
**IGHV8S4**			17,0	104,5	7,0	11,0				32,8	96,5	51,8	103,8	6,7
**IGHV8S7**	2,0		1,7	55,0	52,3	58,8	1,0			**140,0**	219,8	439,3	201,3	68,3
**IGHV8S11**			8,5	35,0	8,5	8,0		1,0		47,3	**68,3**	**38,8**	79,0	5,3
**IGHV9S1**	1,0		73,5	27,0	49,0	36,5				19,3	55,3	77,0	86,7	8,7
**IGHV9S4**			1,0	42,3	2,8	1,0	1,0			120,0	138,0	331,8	166,3	77,0
**IGHV10S1**		1,0	156,3	221,3	166,5	2,0	23,5	2,0	1,0	169,8	334,8	336,8	**183,8**	46,5
**IGHV11S1**		1,0	1,0	40,7	38,3	1,0	1,0			19,7	125,3	182,8	63,0	26,3
**IGHV12S1**	1,5	1,0	72,0	180,3	117,8	1,0	226,0	1,0		208,3	403,8	187,3	213,5	99,0
**IGHV13S1**				47,8	30,7		1,0	1,0	1,0	43,3	116,8	228,0	47,0	60,7

IgT

	GRADE 0							GRADE 2						
	IGHJ1	IGHJ2	IGHJ3	IGHJ4	IGHJ5	IGHJ6	IGHJ7	IGHJ1	IGHJ2	IGHJ3	IGHJ4	IGHJ5	IGHJ6	IGHJ7
**IGHV1S1**	36,0	30,3		1,0	1,7	1,5		**251,3**	107,3		2,0	3,0	1,7	
**IGHV1S4**	59,0	5,0		1,0	1,0			119,8	28,3		1,5	1,0	2,5	
**IGHV1S5**	30,5	18,8		2,0	3,0			**102,8**	91,0		1,0	2,7	3,3	
**IGHV2S1**	27,0	7,8		1,0	1,0	1,0		**61,5**	**28,5**	2,0	2,0	1,5	1,0	1,0
**IGHV4S1**	122,0	45,8		2,0	2,3	1,0		**577,8**	**275,5**		1,7	**6,8**	**6,7**	1,0
**IGHV5S2**	29,0	12,0		2,0	2,0	1,0		182,3	35,5		1,3	3,0	1,3	
**IGHV6S1**	31,5	18,5	1,0	2,0	1,0	1,0		**226,5**	77,0			1,5	1,0	
**IGHV6S4**	10,0	2,0				1,5		**161,5**			1,0	1,0	1,5	
**IGHV8S1**	2,7	15,0		1,0				11,3	9,0			1,0		
**IGHV8S4**	8,0	1,0						10,0	4,0					
**IGHV8S7**	11,3	1,0			1,0	1,0		**80,8**	63,3		1,0	1,0	1,0	
**IGHV8S11**	3,3	2,0			1,0			**9,0**	5,3			1,0		
**IGHV9S1**	162,3	23,3		1,7	1,8	4,0		196,0	78,0		2,0	2,8	4,5	1,0
**IGHV9S4**	19,0	7,3	1,0	1,0		1,0		**130,8**	**40,0**		1,5	1,0	2,0	
**IGHV10S1**	36,3	10,8			1,0			66,5	**51,0**	1,0	1,0	1,0	2,0	1,0
**IGHV11S1**	58,8	26,3		1,0	1,0	1,0		**362,5**	**134,0**		2,0	2,0	2,3	
**IGHV12S1**	30,5	7,5		1,0				100,8	**64,8**	1,0	1,0	2,0	3,0	
**IGHV13S1**	19,0	27,0		2,5		2,0		**148,5**	87,0		1,0		2,0	

Figure S3. Heatmap representation of V-J combinations used. Heatmaps representing the mean number of unique sequences for specific V-J combinations in IgM, IgD and IgT from Grade 0 or Grade 2 kidneys (n=4). Statistical differences (P<0.05) between control and infected groups were analysed with a two-tailed Student's *t* test, and are highlighted in data from Grade 2 fish; significant increases in comparison to values obtained in Grade 0 kidneys are shown in bold while significant increases in comparison to values obtained in Grade 0 kidneys are underlined.

Fig. S4

V-D

IgM

	GRADE 0									GRADE 2								
	IGHD1	IGHD2	IGHD3	IGHD4	IGHD5	IGHD6	IGHD7	IGHD8	IGHD9	IGHD1	IGHD2	IGHD3	IGHD4	IGHD5	IGHD6	IGHD7	IGHD8	IGHD9
**IGHV1S1**	814,3	613,3	342,0	1334,0	791,3	1039,3	766,5	942,8	290,0	676,8	423,8	254,3	1302,8	588,3	426,0	777,3	559,8	212,8
**IGHV1S4**	1420,5	651,0	605,0	1528,0	889,5	1485,3	951,5	2061,3	734,3	<u>510,3</u>	425,3	<u>195,8</u>	937,3	388,8	670,3	742,8	850,0	320,5
**IGHV1S5**	577,5	160,8	318,3	1178,8	615,3	387,8	301,3	244,8	248,3	528,8	88,3	142,0	490,8	346,5	442,5	272,0	387,8	64,5
**IGHV2S1**	927,0	459,8	371,3	1216,3	956,0	767,0	911,8	857,8	498,8	836,0	352,3	336,8	1570,8	811,3	644,5	786,8	<u>442,8</u>	411,5
**IGHV4S1**	505,8	231,0	158,5	719,8	471,0	184,8	941,3	306,0	238,8	**2743,3**	**899,0**	**1000,3**	**3858,0**	**2044,8**	**1380,8**	**1912,5**	**1384,5**	**874,0**
**IGHV5S2**	847,0	510,3	197,5	1224,5	536,5	456,0	668,3	795,8	323,3	945,5	572,5	354,8	1818,3	600,5	640,5	814,5	953,5	434,5
**IGHV6S1**	1309,8	790,5	550,0	2321,3	719,5	1128,0	940,0	1303,8	793,3	892,8	498,0	491,5	1776,0	888,0	649,3	833,3	<u>721,8</u>	545,3
**IGHV6S4**	268,5	124,3	101,3	380,8	154,0	270,3	257,0	516,5	79,5	348,8	363,0	100,0	701,0	253,3	188,0	679,5	503,0	213,5
**IGHV8S1**	236,0	419,8	185,0	409,0	168,8	372,0	244,5	347,5	244,0	254,8	216,8	63,8	324,5	129,3	167,8	351,5	233,8	180,8
**IGHV8S4**	56,8	57,0	28,0	161,0	57,0	67,3	74,3	79,8	24,3	195,3	143,8	69,5	338,3	177,8	153,8	283,0	140,3	93,8
**IGHV8S7**	139,0	84,0	88,3	635,8	247,5	167,8	291,0	154,3	36,0	**532,3**	393,8	**335,3**	1568,3	559,5	705,8	997,0	**472,5**	**318,5**
**IGHV8S11**	32,0	25,8	13,0	60,0	50,3	46,0	43,0	55,8	11,0	152,3	100,3	**49,0**	247,3	144,3	112,8	200,5	103,8	**63,5**
**IGHV9S1**	1686,8	734,0	623,5	2143,5	1471,3	1581,5	1236,0	1378,8	560,5	899,3	393,5	229,0	<u>1047,5</u>	677,0	428,3	824,5	479,5	208,0
**IGHV9S4**	323,0	204,8	195,5	585,3	209,0	323,3	331,0	291,0	74,8	868,5	539,0	248,0	1086,5	539,8	344,3	674,0	656,3	202,5
**IGHV10S1**	2008,3	428,3	787,5	1854,8	1673,8	797,8	1496,5	927,5	515,8	2123,5	616,3	457,5	2757,5	1940,0	525,5	2360,8	1085,0	604,0
**IGHV11S1**	155,8	129,0	75,5	499,0	95,5	391,3	143,5	404,3	55,0	**896,3**	**435,5**	**509,8**	**1665,3**	**764,8**	410,8	**653,8**	879,3	696,5
**IGHV12S1**	523,5	177,5	301,0	1020,3	472,8	192,5	653,3	246,3	191,8	714,0	799,8	410,8	1657,5	629,5	619,0	658,0	396,8	304,8
**IGHV13S1**	102,8	68,8	32,0	241,5	84,0	57,0	114,0	192,3	70,8	**506,5**	**405,8**	**245,5**	**1050,0**	870,8	**273,8**	**848,3**	**869,8**	282,8

IgD

	GRADE 0									GRADE 2								
	IGHD1	IGHD2	IGHD3	IGHD4	IGHD5	IGHD6	IGHD7	IGHD8	IGHD9	IGHD1	IGHD2	IGHD3	IGHD4	IGHD5	IGHD6	IGHD7	IGHD8	IGHD9
**IGHV1S1**	5,8	2,3	3,0	16,8	3,8	2,3	16,0	7,7	3,0	45,8	23,3	**28,8**	69,5	**54,8**	24,0	47,3	81,0	14,0
**IGHV1S4**	1,7	3,0		3,0		1,7	1,5	2,0	1,0	113,5	24,0	11,0	106,0	41,3	22,3	133,0	39,5	39,0
**IGHV1S5**	17,0	26,3	14,7	32,8	36,3	30,7	6,5	46,0	11,5	20,3	2,0	22,0	18,5	11,7	1,0	2,0	2,0	20,5
**IGHV2S1**	207,5	51,5	58,5	162,5	122,0	107,5	84,0	137,3	61,3	415,5	211,8	162,8	**589,8**	368,8	214,3	345,5	227,5	145,5
**IGHV4S1**	9,8	1,0	3,0	9,0	7,7	4,5	5,3	7,7	1,3	21,5	9,3	6,0	28,8	16,3	8,8	12,0	8,3	8,5
**IGHV5S2**	1,7	1,0		4,3	2,0	1,5	5,5	17,0	1,0	**32,0**	26,0	5,0	65,5	28,3	25,8	43,3	23,8	5,3
**IGHV6S1**	1,3	1,0	1,0	1,5	1,0	1,0	1,0	53,0	1,0	20,3	18,0	63,0	43,8	32,3	16,8	18,0	13,8	23,0
**IGHV6S4**	3,0	2,5		4,7	55,0	1,0	4,0	9,0	1,0	7,8	5,0	1,0	12,0	12,3	5,3	25,5	4,3	3,5
**IGHV8S1**	41,0	2,0	4,5	13,0	18,0	1,0	1,0			58,5	19,3	5,0	24,5	15,8	1,8	74,3	35,5	27,7
**IGHV8S4**	6,3	18,0	5,0	47,5	2,0	2,7	6,0	1,5	10,0	28,3	19,8	16,5	102,8	14,3	8,5	32,0	49,5	13,0
**IGHV8S7**	5,3	10,7	16,0	139,3	2,5	4,0	2,0	4,0	2,0	93,5	71,0	58,8	322,8	167,0	79,3	91,3	61,8	61,8
**IGHV8S11**	4,0	10,0	1,0	12,7		2,7	10,0	2,0		24,8	15,3	13,0	78,0	21,3	4,8	23,3	40,0	9,0
**IGHV9S1**	28,5	4,5	14,5	29,5	41,7	30,0	25,0	24,8	4,5	27,3	23,3	7,0	63,0	22,3	18,8	23,5	32,3	14,0
**IGHV9S4**	2,0	2,0		4,8	1,7	3,8	1,0	44,5	1,5	68,3	67,3	42,0	210,3	81,8	**50,7**	63,3	108,7	67,5
**IGHV10S1**	72,3	11,0	16,0	130,3	170,0	42,3	52,0	36,3	45,3	**192,5**	13,0	25,0	236,5	142,3	35,8	264,3	77,0	50,8
**IGHV11S1**	5,5	1,0	14,5	34,0	3,0	56,5	2,0	3,7	1,7	40,0	28,5	48,3	106,8	24,5	22,8	38,3	50,0	24,7
**IGHV12S1**	51,3	11,3	39,7	100,5	43,7	92,7	39,5	35,0	57,0	132,3	44,0	107,8	263,5	126,8	112,8	98,0	50,8	87,5
**IGHV13S1**	22,0	3,0	2,0	11,3	84,0	2,3	4,0	17,7	1,3	38,5	48,7	27,0	108,8	43,3	42,5	42,0	65,0	23,3

IgT

	GRADE 0									GRADE 2								
	IGHD1	IGHD2	IGHD3	IGHD4	IGHD5	IGHD6	IGHD7	IGHD8	IGHD9	IGHD1	IGHD2	IGHD3	IGHD4	IGHD5	IGHD6	IGHD7	IGHD8	IGHD9
**IGHV1S1**	7,0	16,3	17,5	2,0	12,0	11,3		1,7	2,3	68,3	**74,5**	**153,3**	7,3	16,3	6,8	3,3	**5,5**	**9,8**
**IGHV1S4**	84,0	5,8	10,8	1,0	2,0	1,7	1,0	1,5	1,3	21,0	37,0	72,0	6,0	7,3	3,0	1,3	5,0	3,0
**IGHV1S5**	3,3	12,8	23,3	4,5	5,3	3,0	3,0	3,5	2,7	**35,0**	**75,8**	50,5	8,3	21,0	10,3	6,0	4,0	6,0
**IGHV2S1**	8,8	7,3	13,5	1,0	2,5	1,5	1,0	1,0	2,5	**18,8**	20,0	**39,5**	3,8	6,3	2,3	1,3	1,5	2,5
**IGHV4S1**	56,5	28,5	61,0	2,0	9,0	4,0	3,3	1,8	6,5	186,5	**193,0**	**331,5**	**26,0**	45,5	**16,8**	18,8	**20,8**	18,3
**IGHV5S2**	5,5	24,5	15,5		5,0	2,0	3,0	3,5	1,3	**34,0**	76,0	**74,0**	6,7	8,5	5,3	3,0	7,0	8,7
**IGHV6S1**	31,3	2,0	14,0	1,0	2,0	2,0	1,0	1,0	1,0	**106,5**	33,0	**133,5**	2,5	**5,8**	3,3	3,0	7,0	6,5
**IGHV6S4**	4,5	3,0	5,7	1,5	2,0	1,0		1,0		44,3	49,3	**61,8**	8,8	8,5	7,0	8,5	3,0	5,8
**IGHV8S1**	1,0	2,7	12,0		2,0			1,0		9,3	6,0	5,7	1,0	1,7		1,0	2,0	1,5
**IGHV8S4**	3,0	1,5	4,5				1,0	1,0		3,3	4,5	6,5	1,0	1,0	1,0	1,0		
**IGHV8S7**	4,5	2,5	6,0					1,0		**69,3**	14,0	**46,8**	3,0	4,0	10,3	1,0	1,7	3,7
**IGHV8S11**	1,7	1,0	1,3				1,0	1,0		3,5	3,8	**5,3**		4,0	1,5		1,0	2,0
**IGHV9S1**	16,8	56,3	68,8	4,8	20,3	4,5	7,3	1,7	10,0	63,5	71,0	86,3	5,5	26,0	6,0	5,5	3,5	5,0
**IGHV9S4**	7,7	5,7	7,0	2,3	2,0	1,5	1,0	1,0	1,5	34,0	**50,8**	39,8	11,5	9,0	3,8	4,5	5,0	**7,7**
**IGHV10S1**	3,8	14,0	23,5	1,3	2,7	1,5	2,0	1,0	1,5	**15,3**	**35,3**	38,3	**3,5**	10,5	**6,3**	2,5	2,0	5,3
**IGHV11S1**	6,5	21,8	45,5	5,0	1,5	1,5	1,0	1,3	16,0	**117,0**	112,0	**173,8**	19,3	17,3	13,3	7,7	17,3	16,0
**IGHV12S1**	4,3	6,0	20,3	1,0	4,0	2,0	1,0	1,3	2,0	29,8	42,0	67,5	12,3	5,5	4,3	**3,5**	2,0	6,0
**IGHV13S1**	10,0	3,3	23,5	2,5	4,3	2,0	1,0	17,0	1,0	42,3	23,3	**121,0**	11,8	12,8	2,5	2,7	16,3	3,0

Figure S4. Heatmap representation of V-D combinations used. Heatmaps representing the mean number of unique sequences for specific V-D combinations in IgM, IgD and IgT from Grade 0 or Grade 2 kidneys (n=4). Statistical differences (P<0.05) between control and infected groups were analysed with a two-tailed Student's *t* test, and are highlighted in data from Grade 2 fish; significant increases in comparison to values obtained in Grade 0 kidneys are shown in bold while significant increases in comparison to values obtained in Grade 0 kidneys are underlined.

Fig. S5

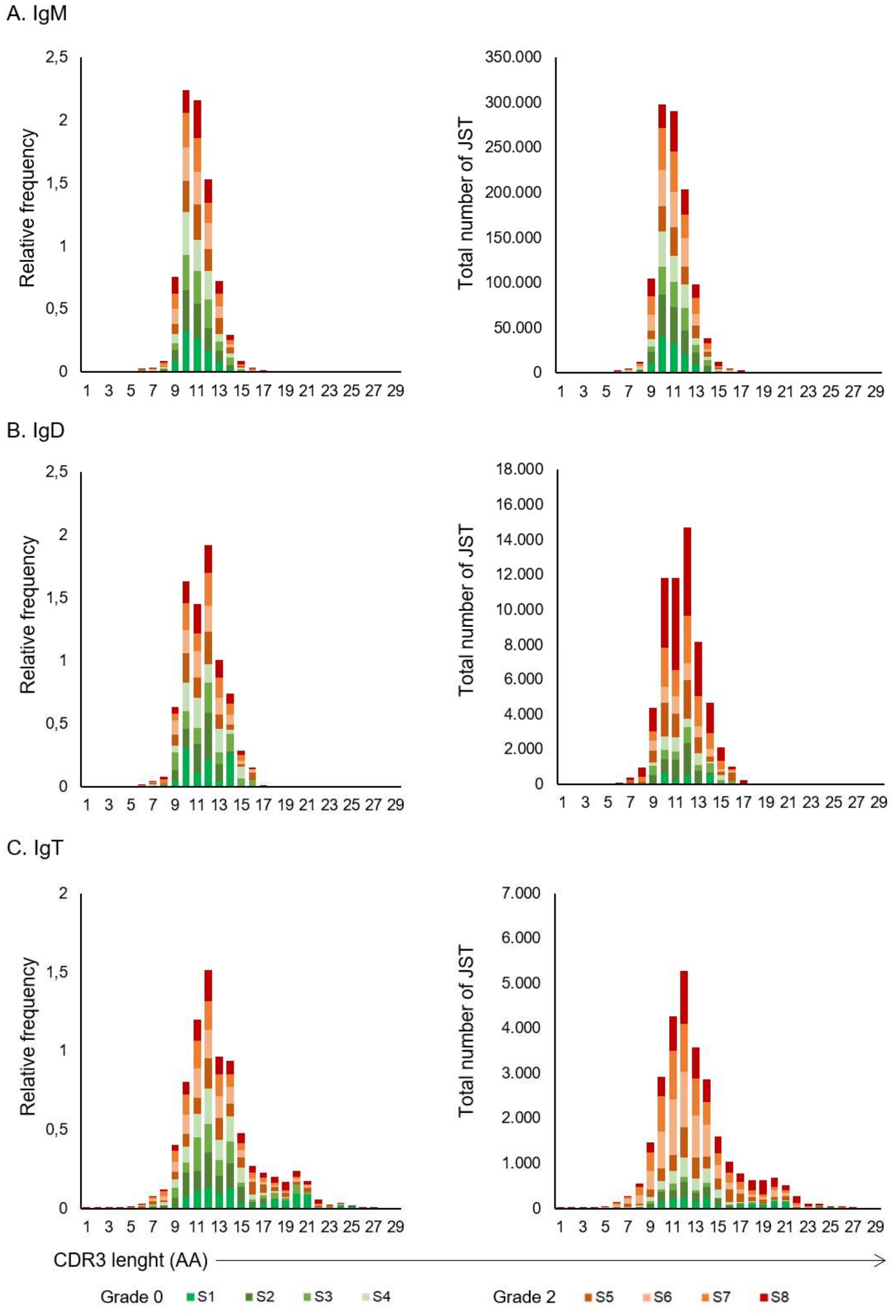

Figure S5. Deep sequencing derived CDR3 length distributions in PKD. Bar charts represent the virtual spectratype compute from the unique sequences datasets for IgM (A), IgD (B) and IgT (C). Bars show the relative frequency or total number of JST associated to a specific CDR3 length, considering the number of amino acids (AA), for each individual studied.
